# Proteomic Analysis of Proteins Surrounding Occludin and Claudin-4 Reveals Their Proximity to Signaling and Trafficking Networks

**DOI:** 10.1371/journal.pone.0117074

**Published:** 2015-03-19

**Authors:** Karin Fredriksson, Christina M. Van Itallie, Angel Aponte, Marjan Gucek, Amber J. Tietgens, James M. Anderson

**Affiliations:** 1 Laboratory of Tight Junction Structure and Function, NHLBI, National Institutes of Health, Bethesda, MD, United States of America; 2 Proteomics Core Facility, NHLBI, National Institutes of Health, Bethesda, MD, United States of America; SRI International, UNITED STATES

## Abstract

Tight junctions are complex membrane structures that regulate paracellular movement of material across epithelia and play a role in cell polarity, signaling and cytoskeletal organization. In order to expand knowledge of the tight junction proteome, we used biotin ligase (BioID) fused to occludin and claudin-4 to biotinylate their proximal proteins in cultured MDCK II epithelial cells. We then purified the biotinylated proteins on streptavidin resin and identified them by mass spectrometry. Proteins were ranked by relative abundance of recovery by mass spectrometry, placed in functional categories, and compared not only among the N- and C- termini of occludin and the N-terminus of claudin-4, but also with our published inventory of proteins proximal to the adherens junction protein E-cadherin and the tight junction protein ZO-1. When proteomic results were analyzed, the relative distribution among functional categories was similar between occludin and claudin-4 proximal proteins. Apart from already known tight junction- proteins, occludin and claudin-4 proximal proteins were enriched in signaling and trafficking proteins, especially endocytic trafficking proteins. However there were significant differences in the specific proteins comprising the functional categories near each of the tagging proteins, revealing spatial compartmentalization within the junction complex. Taken together, these results expand the inventory of known and unknown proteins at the tight junction to inform future studies of the organization and physiology of this complex structure.

## Introduction

Tight junctions (TJs) are localized at the apical end of the lateral plasma membrane of epithelial cells and form charge- and size-selective barriers that regulate paracellular movement of ions and solutes between the apical- and basolateral side of the epithelial cell layers [1]. TJs also function in cell polarity [2] and cytoskeletal regulation [3]. About 40 proteins have been localized to the TJ to date [4], for example, the scaffolding proteins *Zonula Occludens-1* (ZO-1), *ZO-2* and ZO-3 [5], and the transmembrane barrier proteins occludin (Ocln) [6], and claudins [7–9]. However, the list of identified TJ-associated proteins is likely to be incomplete. To expand the inventory of TJ proteins, we recently used biotin ligase fusion proteins to identify proteins proximal to the N- or C-termini of ZO-1 [10]. The proteins identified in this analysis included numerous previously identified TJ proteins and in addition a variety of trafficking, signaling, cytoskeletal and polarity proteins. Although many proteins were found in proteomic analyses from both fusion proteins, some proteins were uniquely identified as proximal to either the N- or the C-terminus of ZO-1 [10]. Further, comparison of ZO-1 proximal proteins with a recently generated list of proteins proximal to the adherens junction (AJ) protein, E-cadherin, revealed relatively little overlap, suggesting that the biotin ligase tagging method has a high degree of spatial resolution [11]. Thus, to gain further insights into TJ architecture we applied this method to the transmembrane proteins Ocln and claudin-4 (Cldn4); with the goal of comparing their proximal proteomes with those of ZO-1 and E-cadherin.

Occludin, a 65 kDa tetraspan protein was the first transmembrane protein identified at the TJ more than twenty years ago by Furuse et al. [6]. Although Ocln is a nearly invariant constituent of TJ, its functional role at the TJ is still not fully understood. Overexpression of Ocln in MDCK II cells leads to increased transepithelial resistance (TER) [12], whereas Ocln KO mice display an almost normal phenotype [13]. By itself, Ocln does not form the fibrils that characterize the TJ in freeze fracture electron microscopy, however it does co-polymerize with claudins in these strands [7]. The C-terminus of Ocln has been shown to bind ZO-1, subsequently mediating its intracellular trafficking to the lateral plasma membrane and TJs [14]. Ocln phosphorylation has been associated with concentration at the TJ [15] and Ocln extracellular loops and one transmembrane domain have been shown to contribute to its TJ localization and stability [16–18]. Although the role of Ocln in paracellular barrier function is yet not fully understood, numerous studies implicated functions in junctional signaling [14,19–23] and trafficking pathways [24–27]. Taken together, these previous findings suggested that proteomic analysis of proteins proximal to both the N- and the C-terminus of Ocln might help elucidate relevant junctional signaling, trafficking and cytoskeletal proteins.

The main barrier forming proteins of the TJ are the 24 members of the claudin family of proteins [28]. Claudins are the main structural elements of the TJ and varying claudin composition specifies the barrier properties of epithelia in different organs and tissues [28–32]. Like Ocln, claudins contain four transmembrane helices; however, claudins are much smaller with molecular masses between 21–28 kDa [29]. Overexpression of Cldn4 in MDCK II cells increases TER by selectively decreasing Na^+^ permeability (*P*_Na_) over Cl^-^ permeability (*P*_Cl_), and also increases the number of freeze-fracture fibrils [33]. However, like many other claudins, Cldn4 distribution is not limited to the TJ but is also localized along the lateral membrane [34]. Proteomic analysis of proteins proximal to Cldn4 would thus be expected to reveal TJ and trafficking proteins, as well as relevant lateral membrane and cytoskeletal proteins. As a caveat, this method does not allow us to discriminate between proteins proximal to Ocln and Cldn4 at the TJ versus on the lateral membrane.

Proteins identified in this study included many known TJ and AJ proteins. In addition, we also found signaling, trafficking, cell-adhesion, cytoskeletal, and polarity proteins that deserve further investigation for their putative roles in different aspects of junction regulation, including cytoskeletal organization, cell-cell and cell-matrix adhesions, cell migration and proliferation. A number of proteins were biotinylated exclusively or predominantly by biotin ligase fused to either the N- or C-terminus of Ocln and/or the N-terminus of Cldn4, indicating the spatial specificity of this method. This inventory of Ocln and Cldn4 neighboring proteins may lead to new discoveries and insights into the regulation and function of the TJ.

## Materials and Methods

### Constructs

Myc-biotin ligase plasmid (pcDNA3.1 mycBioID) was a gift of Kyle Roux (Addgene, Cambridge, MA; plasmid 35700) [35]; the Myc-biotin ligase insert was excised and subcloned into pTRE2hyg (BD Biosciences, San Diego, CA); full-length human occludin and claudin-4 was subcloned 3′ to the biotin ligase (BL) coding region (BL-Ocln, BL-Cldn4) using the In-Fusion PCR-based cloning kit (BD Biosciences). Ocln-BL was made by cloning full-length human Ocln 5′ to the biotin ligase coding sequence and moving the Myc tag to the C-terminal end of the fusion protein as previously described [10]. The C-terminal PDZ-binding motif of claudins is required for proper localization, and therefore we fused biotin ligase only with the N-terminus of Cldn4 [36].

pEGFP-C1-RNtre was a gift of Letizia Lanzetti, Torino, Italy; the insert was excised and subcloned into pTRE2hyg (BD Biosciences, San Diego, CA). pCMV6-AC-GFP-FLRT2 (MG209836) was purchased from Origene (Rockville, MD) and was subcloned into pEGFP-N1 (U557622, Clontech, Palo Alto, CA). pOTB7-PLLP (clone id: 3633345) was purchased from Thermo Scientific and was subcloned into pEGFP-C1 (U55763, Clontech). In-fusion primers used can be found in S1 Table. All constructs were verified by DNA sequencing (ACGT Inc., Wheeling, IL) using specific primers.

### Cell Culture, Immunoblots, and Immunofluorescence

Tet-off MDCK II cells (BD Biosciences) were cultured under standard conditions in DMEM (4.5 g/liter glucose), 10% fetal bovine serum, and penicillin/streptomycin. Transfections with BL-Ocln, Ocln-BL and BL-Cldn4 encoding plasmids were performed by Nucleofection (Lonza, Allendale, NJ). Stable antibiotic-resistant Ocln and Cldn4 biotin ligase fusion protein expressing MDCK II cells were selected using hygromycin (250 μg/ml). Stable clones were screened for transgene expression by immunoblot as previously described [10]. Transfections of MDCKII cells with GFP-FLRT2, GFP-PLLP and EGFP-RNtre were also performed by Nucleofection followed by Hygromycin selection and stable clones screened by immunoblot. Antibodies used were rabbit anti-Mark3 (ab52626), rabbit anti-GFP (ab290), abcam, Cambridge, MA, and mouse anti-occludin (catalog no. 33–1500), mouse and rabbit anti-claudin-4 (32–9488, 36–4800) and mouse anti-ZO-1 (33–9100), all from Life Technologies (Carlsbad, CA). Rat anti-ZO-1 used was R40.76 [37]. Secondary antibodies for immunoblot were from Rockland (Gilbertsville, PA), and secondary antibodies for immunofluorescence were from Jackson Immunoresearch (West Grove, PA) except for Streptavidin 568 (Life Technologies). Immunofluorescence was performed as described previously [38]. Cells were fixed in 100% ice-cold ethanol. Images were taken using a Zeiss LSM UV confocal microscope, ×40 or x63 oil lens, and images were generated using Zeiss Zen software. Montages were generated using Adobe Photoshop or Windows Paint and PowerPoint.

### Purification of Biotinylated Proteins for Mass Spectrometry

Purification of biotinylated proteins from MDCK II cells stably expressing transgenes was performed as previously described [10,11]. Briefly, cells were induced to express transgenes, incubated with 50 μM biotin for 15–17h, lysed, and biotinylated proteins were purified on streptavidin resin. Eluted proteins were subjected to SDS-PAGE, and gels were stained briefly with SimplyBlue Safe Stain (Life Technologies) before further processing to prepare samples for mass spectrometry.

### Mass Spectrometry, MASCOT Database Search and Data Analysis

Liquid chromatography tandem mass spectrometry was performed using an Eksigent nanoLC-Ultra 1D Plus system (Dublin, CA) coupled to an LTQ Orbitrap Elite mass spectrometer (Thermo Fisher Scientific) using collision- induced dissociation fragmentation. Peptides were first loaded onto a Zorbax 300SB-C18 trap column (Agilent, Palo Alto, CA) at a flow rate of 6 μl/min for 6 min and then separated on a reversed-phase PicoFrit analytical column (New Objective, Woburn, MA) using a 40-min linear gradient of 5–40% acetonitrile in 0.1% formic acid at a flow rate of 250 nl/min. LTQ- Orbitrap Elite settings were as follows: spray voltage, 1.5 kV; full MS mass range, *m*/*z* 300–2000. The LTQ-Orbitrap Elite was operated in a data-dependent mode (*i*.*e*. one MS1 high resolution (60,000) scan for precursor ions followed by six data- dependent MS2 scans for precursor ions above a threshold ion count of 500 with collision energy of 35%).

The raw file generated from the LTQ Orbitrap Elite was analyzed using Proteome Discoverer version 1.3 software (Thermo Fisher Scientific, LLC). Data was submitted to Mascot v2.4 (Matrix Sciences) search engine with the following search criteria: database, National Center for Biotechnology Information (NCBI) RefSeq taxonomy (*Canis lupus familiaris*, *(dog))*; enzyme, trypsin; miscleavages, 2; variable modifications, oxidation (M), deamidation (NQ), acetyl (protein N-Term), Biotin (N-term), Biotin (K); fixed modification, carbamidomethyl (C); MS peptide tolerance 20 ppm; MS/MS tolerance as 0.8 Da. Post-database search, the peptides were filtered for a false discovery rate of 1% (using target decoy database) and rank 1 peptides (unique to one protein).

All samples were analyzed in triplicates from three independent experiments. Protein inclusion criterion required a protein be present in at least two of the three experiments. After proteins were compiled, keratins, histones, and endogenously biotinylated carboxylases were discarded before calculating the total peptide spectrum match (PSM) for each individual experiment and the normalized PSM for each protein. The average normalized PSM/Observable Peptide Number (OPN) (av n-PSM/OPN) was then calculated as previously described [11]. Ribosomal proteins were removed after the total PSM and normalized PSM for individual proteins in each run was calculated (ribosomal proteins are reported below the other identified proteins for each biotin ligase construct in S2 and S3 Tables). As previously described we also removed all proteins that were less than three times enriched when labeled by biotin ligase Ocln or Cldn4, as compared to the biotin ligase alone, before further functional analysis [11]. The complete protein lists can be found in S2 Table and the enriched protein lists in S3 Table. Only the top 150 proteins enriched around Ocln and Cldn4 were included in further analysis (S4 Table). Primarily UniProt descriptors [39], but also primary literature searches were used to classify proteins into functional categories. The S2 and S3 Tables are organized with the most abundant protein at the top and then in descending order as calculated by the average normalized PSM/Observable Peptide Number. Tables 1–8 and S4 Table are organized relative to the proteomic rank order list generated by BL-Ocln. This means that proteins highly enriched in the Ocln-BL and/or BL-Cldn4 proteomes, but not in the BL-Ocln, are found below BL-Ocln in Tables 1–8 and S4 Table. Proteins enriched in the ZO-1 and E-cad proteomes, that were not present in lists from Ocln and Cldn4 biotin ligase constructs, are not listed.

**Table 1:** Enriched tight junction (TJ) and adherens junction (AJ) proteins tagged by biotin ligase fused to occludin and claudin-4.

Accession	Name	Localization/Function-Tight Junction, Adherens Junction	OCLN N	OCLN C	CLD4 N	ZO-1 N	ZO-1 C	E-CAD	Reference
50978954	Occludin	Integral membrane protein of TJ.	252.2	99.4	21.4	29.6	ND	1.6	[6]
345795509	Coxsackievirus and adenovirus receptor homolog	Transmembrane protein essential for TJ integrity.	28.2	19.3	47.2	45.4	5.4	5.1	[93]
50978966	Tight junction protein ZO-2	Scaffolding protein localized at TJ. Also localized in the nucleus.	18.8	30.5	ND	72.9	33.9	1.9	[94]
55741803	Tight junction protein ZO-1	Scaffolding protein localized at TJ.	17.1	28.6	ND	273.7	286.5	6.9	[95]
345796449	Claudin-16	Integral membrane protein of TJ.	16.1	4.4	20.8	ND	ND	ND	[96]
50978770	Claudin-3	Integral membrane protein of TJ.	15.2	1.37	36.6	59.7	ND	ND	[9]
74003604	Claudin-1	Integral membrane protein of TJ.	14.4	6.2	25.7	17.4	ND	ND	[7,8]
73982228	Catenin delta-1	AJ protein implicated both in cell transformation by SRC and in receptor signaling. May also be involved in Wnt signaling.	12.8	14.4	40.3	ND	ND	39.6	
73981482	Membrane-associated guanylate kinase, WW and PDZ domain-containing protein 3	Scaffolding protein, localized at TJ.	11.1	15.6	4.0	9.0	ND	ND	[97]
50978772	Claudin-2	Integral membrane protein of TJ.	10.9	3.9	8.8	35.4	ND	ND	[7,8]
50950163	Band 4.1-like protein 5	Positioning of TJ during the establishment of polarity.	7.2	7.0	9.7	ND	ND	1.5	[98]
345784504	Afadin, Af6, MLLT4	Involved in various types of cell motility, AJ.	6.6	9.2	5.2	6.0	ND	11.5	[99,100]
345793058	Syntenin-1	Adapter protein. Couples syndecans to cytoskeletal proteins at AJ.	6.3	2.7	ND	ND	ND	ND	[101]
359319613	Claudin-4	Integral membrane protein of TJ.	5.8	(1.5)	15.6	8.2	ND	ND	[33,43]
359322181	Membrane-associated guanylate kinase, WW and PDZ domain-containing protein 1	Scaffolding protein, localized at TJ.	4.9	7.9	(0.9)	3.0	0.4	(0.2)	[102]
73947369	Poliovirus receptor-related protein 2	Immunoglobulin-like cell-cell adhesion molecule at AJs. Is associated with e-cad through afadin and catenins, which connect to the actin cytoskeleton.	4.3	2.8	17.1	3.9	ND	1.3	[103,104]
345797440	Partitioning defective 3 homolog B	TJ protein involved in polarization of epithelial cells.	4.2	4.3	(1.0)	1.4	0.6	(0.7)	[105]
345796147 345796149 345796154	Disks large homolog 1	Multidomain scaffolding protein of the MAGUK family. Has a role in AJ assembly.	3.4	2.5	6.0	ND	ND	2.6	[106]
50978964	Tight junction protein ZO-3	Peripheral membrane protein of TJ.	3.0	1.7	(1.4)	12.0	5.3	ND	[107]
345793331	Partitioning defective 3 homolog	TJ adapter protein involved in asymmetrical cell division and cell polarization.	(2.5)	4.0	(1.0)	0.9	ND	(0.4)	[108]
73948496	Lipolysis-stimulated lipoprotein receptor	Recruits tricellulin to tricellular TJ.	(2.4)	1.6	4.9	1.1	ND	(0.8)	[109]
345778074	Catenin alpha-1	AJ protein.	(2.1)	4.4	(1.0)	ND	ND	192.2	[110,111]
345779559	Protein scribble homolog, partial	Scaffold protein involved in different aspects of polarization. TJ assembly.	(0.8)	2.5	(0.4)	ND	ND	3.2	[112]
345797675	Junctional adhesion molecule A	Single-pass membrane protein localized at TJ in epithelial cells.	(0.6)	ND	4.9	5.9	ND	ND	[113]
73970139	Epithelial cell adhesion molecule	Single-pass lateral membrane protein. Co-localizes with CLDN7.	(0.3)	ND	4.4	ND	ND	ND	[114,115]
345800417	InaD-like protein	Scaffolding protein that regulates protein targeting, cell polarity and integrity of TJ.	(0.4)	1.60	ND	0.6	ND	ND	
212549677	Catenin beta-1	AJ protein. Key downstream component of the canonical Wnt signaling pathway.	ND	1.6	(1.0)	ND	ND	36.5	
345800139	Claudin-7	Integral membrane protein of TJ.	ND	ND	16.0	4.2	ND	ND	[9]

Numbers in the columns for biotin ligase constructs are average normalized PSM/OPNx1000 from 3-fold enriched proteins compared to the biotin ligase alone [11]. PSM is based on peptide fragmentation and subsequent sequencing by collision-induced dissociation (CID) where the same precursor mass can be sequence more than once. Numbers in parenthesis shows that the protein is enriched, however not in the top 150. Not detectable (ND) means that a protein is not enriched. Data from ZO-1 and E-cad are taken from previously published data [10,11]. If a reference is not listed in the far right column, UniProt is the source of protein localization/function. *Italic* proteins fall into more than one functional category, for example exocytosis and endocytosis.

**Table 2:** Enriched signaling proteins tagged by biotin ligase fused to occludin and claudin-4.

Accession	Name	Localization/Function-Signaling	OCLN N	OCLN C	CLD4 N	ZO-1 N	ZO-1 C	E-CAD	Reference
345805018 345805020	Adapter molecule crk	Adapter molecule also known as p38. Participates in the Reelin signaling.	24.6	71.1	ND	17.6	51.6	22.1	
73950745	EF-hand domain-containing protein D2	Negatively regulates the canonical NF-kappa-B-branch.	16.6	ND	ND	ND	ND	ND	
345800829	WW domain-containing oxidoreductase	Inhibits Wnt signaling by sequestering DVL2.	12.4	11.1	(3.4)	8.5	2.6	29.2	
359319518	Segment polarity protein dishevelled homolog DVL-1	May play a role in the signal transduction pathway mediated by multiple Wnt genes.	8.6	5.4	(2.9)	4.8	2.4	ND	
359320259	Na(+)/H(+) exchange regulatory cofactor NHE-RF1	Scaffold, connecting plasma membrane with the cytoskeleton. May enhance Wnt signaling. Regulates phosphate reabsorption in proximal tubules.	7.5	9.5	12.2	ND	ND	ND	
345807425	Ephrin-B1	Binds to Eph receptors leading to bidirectional signaling into neighboring cells.	6.7	3.9	13.9	ND	ND	2.2	
73983369	Muscarinic acetylcholine receptor M4	Mediates various cellular responses, including inhibition of adenylate cyclase.	5.6	ND	3.5	ND	ND	ND	
73955537	Segment polarity protein dishevelled homolog DVL-2	Involved in canonical and non-canonical Wnt signaling, promotes internalization and degradation of frizzled.	4.9	5.2	ND	8.6	2.1	2.3	
345796663	Segment polarity protein dishevelled homolog DVL-3	May play a role in the signal transduction pathway mediated by multiple Wnt genes.	4.7	5.0	ND	6.8	ND	ND	
73994955	Merlin	Regulator of Hippo/SWH signaling. Tumor suppressor.	4.3	3.6	ND	ND	ND	ND	
57095034	Protein FAM83B	A key intermediary in EGFR/RAS/MAPK signaling.	4.3	(0.3)	6.2	0.3	ND	(0.7)	[116]
345777622	SH2 domain-containing adapter protein B	Adapter protein linking activated receptors to downstream signaling components.	3.8	3.4	(2.5)	ND	ND	ND	
73997796	Tubby-related protein 3	Negative regulator of Shh signaling transduction pathway.	3.8	6.7	6.8	ND	ND	ND	
73968671	Fibroblast growth factor receptor substrate 2	Adapter protein linking activated FGR and NGF receptors to downstream signaling pathways.	3.0	3.4	1.5	ND	ND	(0.7)	
345804663	G-protein coupled receptor family C group 5 member C	Coupled receptor for a interaction between retinoid and G-protein signaling pathways.	(2.6)	ND	3.6	ND	ND	ND	
345793416 73948705 345793418	Abl interactor 1	Involved in cytoskeletal reorganization and EGFR signaling. May negatively regulate of cell growth and transformation.	(2.1)	2.3	10.0	0.4	ND	ND	
73948986	Signal transducing adapter molecule 1	Involved in intracellular signal transduction mediated by cytokines and growth factors.	(1.3)	2.4	ND	ND	ND	5.5	
57044069	Wiskott-Aldrich syndrome protein family member 2	Signals from tyrosine kinase receptors and small GTPases to the actin cytoskeleton.	(1.1)	(0.4)	4.2	ND	ND	ND	
345791046	TBC1 domain family member 10A	Acts as GTPase-activating protein for RAB27A.	(0.9)	(0.7)	4.3	ND	ND	ND	
345792359	Epidermal growth factor receptor kinase substrate 8	May play a role in membrane ruffling and remodeling of the actin cytoskeleton.	ND	15.0	22.5	ND	ND	(0.9)	
73995905	V-crk sarcoma virus CT10 oncogene homolog (avian)-like	Adapter protein involved in several signaling pathways.	ND	13.7	ND	14.0	17.8	ND	
345784160	GRB2-associated-binding protein 1	Signaling adapter triggered by receptor-type kinases. Acts in FGFR1 signaling.	ND	6.5	ND	ND	ND	1.1	
73990204	Cytoplasmic protein NCK1	Role in transcriptional activation in response to activated Ras. Role in cell adhesion and migration through ephrin.	ND	5.4	ND	ND	ND	ND	
345787801	Switch-associated protein 70	Mediates signaling of membrane ruffling. Regulates the actin cytoskeleton as an effector or adapter protein	ND	3.9	ND	ND	ND	ND	
345790868	BRCA1-associated protein	Negatively regulates MAP kinase activation. Also acts as a Ras responsive E3 ubiquitin ligase. Act as a cytoplasmic retention protein.	ND	2.6	ND	ND	ND	ND	
359320319	Tax1-binding protein 3-like	Inhibits TNF-induced apoptosis. Play a role in the pro-inflammatory cytokine IL-1 signaling.	ND	2.5	ND	ND	ND	1.2	
345796461	Coiled-coil domain-containing protein 50	Involved in EGFR signaling.	ND	1.9	ND	ND	ND	1.2	
73946134	A-kinase anchor protein 12	Anchoring protein that mediates the subcellular compartments of PKA and PKC. May be part of the cortical cytoskeleton.	ND	ND	8.7	ND	ND	ND	
73992442	Tumor protein D54 isoform 1	Vesicle transport, signaling and tumor progression.	ND	ND	5.5	ND	ND	3.2	
359319375	Tumor-associated calcium signal transducer 2	EPCAM family member. May function as a growth factor receptor.	ND	ND	5.3	ND	ND	ND	[63]
73988027	Ragulator complex protein LAMTOR1	Involved in amino acid sensing and activation of mTORC1. Promotes cell growth in response to growth factors.	ND	ND	4.5	ND	ND	ND	

**Table 3:** Enriched kinases and phosphatases tagged by biotin ligase fused to occludin and claudin-4.

Accession	Name	Localization/Function-Kinases	OCLN N	OCLN C	CLD4 N	ZO-1 N	ZO-1 C	E-CAD
73964123 73964085	MAP/microtubule affinity-regulating kinase 3	Involved in the specific phosphorylation of microtubule-associated proteins for tau.	24.8	17.9	4.6	1.9	ND	3.5
73962894	Mitogen-activated protein kinase kinase kinase kinase 5	Component of the MAP kinase pathway. Signaling for cell fate such determination.	23.3	ND	ND	ND	ND	ND
359321812	Serine/threonine-protein kinase MARK2	Involved in cell polarity and microtubule dynamics regulation. Positive regulator of the Wnt signaling.	16.1	15.0	10.2	4.0	ND	4.0
73953687	Casein kinase I isoform alpha	Participates in Wnt signaling.	15.6	8.8	ND	23.9	ND	ND
359320638	Mitogen-activated protein kinase kinase kinase kinase 4	Serine/threonine kinase that may play a role in the response to environmental stress and cytokines such as TNF-alpha. Appears to act upstream of the JUN N-terminal pathway	9.4	7.5	(2.6)	ND	ND	ND
73998466 345792771	STE20-like kinase	Mediates apoptosis and actin stress fiber dissolution.	8.2	2.9	19.8	ND	ND	ND
345782253	Mitogen-activated protein kinase kinase kinase kinase 3	An essential component of the MAP kinase signal transduction pathway.	7.9	8.4	ND	ND	ND	ND
73954046	Serine/threonine-protein kinase 10	Phosphorylates MSN and possibly PLK1. A negative regulator of MAP3K1/MEKK1.	7.8	ND	8.5	ND	ND	ND
359319430	Misshapen-like kinase 1	Negative regulator of Ras-related Rap2-mediated signaling, can activate the JNK and MAPK14/p38 pathways.	7.5	3.8	4.0	ND	ND	ND
50979026	Tyrosine-protein kinase Yes	Regulation of cell growth and survival, apoptosis, cell-cell adhesion, cytoskeleton remodeling, and differentiation.	4.6	ND	9.7	ND	ND	ND
345804687	Casein kinase I isoform delta	Participates in Wnt signaling.	4.5	5.2	ND	1.1	ND	ND
73969119	Casein kinase I isoform epsilon	Participates in Wnt signaling.	4.4	4.4	ND	ND	ND	ND
73947690	Serine/threonine-protein kinase PAK 4	Acts in a variety of signaling pathways including cytoskeleton regulation, cell migration, growth, proliferation and survival.	3.9	ND	ND	1.5	ND	ND
73999120	Tyrosine-protein kinase Lyn	Transmits signals from receptors. Regulates immune responses, hematopoiesis, responses to growth factors and cytokines, integrin signaling, DNA damage and genotoxic agents. Primarily as negative regulator, but also as an activator.	3.5	ND	12.1	ND	ND	ND
73960795	Serine/threonine-protein kinase MRCK alpha	Downstream effector of CDC42 and plays a role in the regulation of cytoskeleton reorganization and cell migration.	3.1	1.6	(0.6)	ND	ND	ND
73991940	Proto-oncogene tyrosine-protein kinase Src	Participates in signaling pathways controlling a diverse spectrum of biological activities.	2.8	(0.8)	7.7	ND	ND	(0.2)
73950854	Ephrin type-A receptor 2	Binds ephrin-A ligands on adjacent cells, leading to bidirectional signaling into neighboring cells.	(1.5)	(0.2)	8.3	ND	ND	(0.6)
345782271	Kinase D-interacting substrate of 220 kDa	Participates in Wnt signaling.	(1.4)	2.3	ND	ND	ND	1.1
345781192	Ephrin type-A receptor 1	Receptor tyrosine kinase. Binding to ephrin-A leads to bidirectional signaling into neighboring cells. Induces cell attachment inhibiting cell spreading and motility. Plays a role in angiogenesis and regulates cell proliferation.	(1.0)	ND	10.6	ND	ND	ND
345790915	Serine/threonine-protein kinase TAO3	A regulator of the p38/MAPK14 MAPK cascade and the MAPK8/JNK cascade. Inhibits basal activity of MAPK8/JNK cascade and diminishes its activation in response EGF.	ND	ND	5.5	ND	ND	ND

Accession	Name	Localization/Function-Phosphatases	OCLN N	OCLN C	CLD4 N	ZO-1 N	ZO-1 C	E-CAD
73983267	Receptor-type tyrosine-protein phosphatase eta	Dephosphorylates many regulatory proteins. Plays a role in cell adhesion, migration, proliferation and differentiation. Positive regulator of cell-matrix adhesion, endothelial cell survival and VEGF-induced SRC and AKT activation. Negative regulator of cell proliferation, PDGF-stimulated cell migration, EGFR signaling etc. Enhances the barrier function of epithelial junctions during reassembly.	3.5	(1.3)	4.9	ND	ND	ND
345795711	Tyrosine-protein phosphatase non-receptor type 13	Negatively regulates FAS-induced apoptosis and NGFR-mediated pro-apoptotic signaling. Localizes at regions of large actin turnover.	3.0	2.8	ND	ND	ND	ND
50978728	Serine/threonine-protein phosphatase PP1-alpha catalytic subunit	Associates with over 200 regulatory proteins to form highly specific holoenzymes which dephosphorylate hundreds of biological targets. PP1 is essential for cell division, regulates of glycogen metabolism, muscle contractility and protein synthesis. Regulates ionic conductances and long-term synaptic plasticity.	2.3	ND	ND	2.7	ND	1.2

**Table 4:** Enriched endosomal- and membrane-trafficking proteins tagged by biotin ligase fused to occludin and claudin-4.

Accession	Name	Localization/Function-Endocytosis	OCLN N	OCLN C	CLD4 N	ZO-1 N	ZO-1 C	E-CAD	Reference
*345806493 74010356*	*Synaptobrevin homolog YKT6-like*	*Functions in post-Golgi recycling pathways*. *Acts as a recycling carrier to the cell surface*.	*61*.*0*	*26*.*6*	*78*.*6*	*ND*	*ND*	*ND*	
345800559	Vesicle-associated membrane protein 2	Endosome-Trans golgi. Involved in the targeting and/or fusion of transport vesicles to their target membrane.	58.0	63.1	101.4	ND	ND	17.1	
73980912	Vesicle-associated membrane protein 5	Endocytosis/Golgi. Plasma membrane, intracellular perinuclear and peripheral vesicular structures of myotubes. Trans-Golgi association.	38.1	20.9	64.6	16.3	ND	7.2	
57108129	Synaptosomal-associated protein 23	Vesicle docking and fusion, mainly plasma membrane localization.	19.3	14.6	56.4	ND	ND	15.0	
73949154	USP6 N-terminal like	Receptor trafficking. Inhibits internalization of EGFR.	16.5	20.9	13.4	9.0	ND	5.3	
*73996013*	*Synaptosomal-associated protein 29*	*Exocytosis/Endocytosis*. *Involved in membrane trafficking steps*, *binds to multiple syntaxins*.	*15*.*2*	*17*.*5*	*16*.*0*	*ND*	*ND*	*9*.*3*	*[117]*
359321653	Rab11 family-interacting protein 5-like	Endocytosis. Involved in protein trafficking from apical recycling endosomes to the apical plasma membrane.	10.1	6.5	ND	ND	ND	ND	
50979156	Ras-related protein Rab-7a	Endosome-Lysosome transport. Endosomal maturation, microtubule minus-end/plus-end directed endosomal migration/positioning.	8.5	3.5	56.7	ND	ND	2.1	
73979203 345781540	Rab11 family-interacting protein 1	Translocates with RAB11A from vesicles of the endocytic recycling compartment to the plasma membrane.	8.2	7.6	4.7	ND	ND	ND	
73973432	Ras-related protein Rab-23	Endocytosis. Also plays a role in autophagic vacuole assembly. Mediates pathogen defense by promoting their capture by autophagosomes which merge with lysosomes. Negative regulator of Shh signaling.	7.1	4.2	12.4	ND	ND	ND	
73945522	Syntaxin-7	Endocytosis. Mediates the endocytic trafficking from early endosomes to late endosomes and lysosomes.	5.8	5.6	ND	ND	ND	3.8	
359320868	Sorting nexin-3	Multivesicular body formation. Protein transport between cellular compartments.	4.8	2.7	7.5	ND	ND	1.8	
345797971 74005894	Ras-related protein Rab-7L1	Endocytic trafficking from early endosomes to late endosomes and lysosomes.	4.8	ND	ND	ND	ND	(1.0)	
345792855	Rab11 family-interacting protein 2	Translocates with RAB11A from the vesicles of the endocytic recycling compartment to the plasma membrane.	4.6	1.6	ND	ND	ND	ND	
*311771751*	*Ras-related protein Rab-10*	*Exocytosis/Endocytosis*.	*4*.*0*	*2*.*2*	*6*.*0*	*ND*	*2*.*3*	*ND*	
54792725	Ras-related protein Rab-11A	Endocytic recycling.	3.6	ND	(2.7)	ND	ND	ND	
345798663 73952189	Secretory carrier-associated membrane protein 1	Functions in post-Golgi recycling pathways. Acts as a recycling carrier to the cell surface.	3.4	ND	11.6	ND	ND	ND	
73979956 345782124	Sorting nexin-17	Critical regulator of endosomal recycling.	3.3	(0.8)	ND	ND	ND	ND	
345802882	Syntaxin-6	Endosome-Trans golgi.	2.9	2.0	(3.0)	ND	ND	ND	
73968313	Ras-related protein Rab-5B	Endocytosis. Localized to the plasma membrane and early endosomes.	2.8	2.0	3.4	ND	ND	ND	[118]
73950822	Adaptin ear-binding coat-associated protein 2	Endocytosis. Interacts with the adapter protein complexes AP-1 and AP-2.	(2.7)	4.0	ND	ND	ND	2.1	
50979110	Caveolin-1	A scaffolding protein within caveolar membranes. May regulate CTNNB1-mediated signaling through the Wnt pathway.	(2.7)	(0.9)	5.6	ND	ND	ND	
345781300	Extended synaptotagmin-2	Rapid endocytosis of activated FGF receptor and for functional signaling during Xenopus development.	(2.6)	ND	5.0	ND	ND	ND	
359320658	Protein kinase C and casein kinase substrate in neurons protein 2	Plays a role in caveola-mediated endocytosis.	(2.2)	4.4	ND	ND	ND	ND	
*359320530*	*RAB21*, *member RAS oncogene family*, *partial*	*Endocytosis/ER-Golgi transport*. *Regulates integrin internalization and recycling*. *In polarized cells*, *observed in vesicles in the apical cytoplasm*.	*(1*.*4)*	*(1*.*0)*	*3*.*8*	*ND*	*ND*	*(0*.*8)*	
*345807408*	*Vesicle-associated membrane protein 7*	*Endosome-Lysosome/ER-Golgi/Exocytosis*. *Involved in the targeting and/or fusion of transport vesicles to their target membrane*. *Required for focal exocytosis of late endocytic vesicles during phagosome formation*.	*(0*.*7)*	*ND*	*4*.*3*	*ND*	*ND*	*ND*	
359322148	Amyloid beta A4 precursor protein-binding family A member 3	Endocytic recycling and cytoskeleton remodeling. May modulate vesicle budding and uncoating within the Golgi apparatus. Contributes to the regulation of dendritic branching and filopodia extension. Interacts with RAB11FIP3, RAB11FIP4 and USP6.	ND	ND	4.8	ND	ND	ND	

**Table 5:** Enriched clathrin-dependent endocytosis and exocytosis/transcytosis trafficking proteins tagged by biotin ligase fused to occludin and claudin-4.

Accession	Name	Localization/Function-Exocytosis/Transcytosis	OCLN N	OCLN C	CLD4 N	ZO-1 N	ZO-1 C	E-CAD	Reference
*73996013*	*Synaptosomal-associated protein 29*	*Exocytosis/Endocytosis*. *Involved in multiple membrane trafficking steps*, *binds tightly to multiple syntaxins*.	*15*.*2*	*18*	*16*.*0*	*ND*	*ND*	*9*.*3*	*[117]*
73980368	Vesicle-associated membrane protein 8	Exocytosis. Involved in the targeting and/or fusion of transport vesicles to their target membrane. Involved in the homotypic fusion of early and late endosomes.	10.8	7.4	6.8	ND	ND	1.5	
73962778	Syntaxin-binding protein 6	Exocytosis. May modulate the formation of functional SNARE complexes and exocytosis.	4.0	1.9	5.3	ND	ND	ND	
*311771751*	*Ras-related protein Rab-10*	*Exocytosis/Endocytosis*.	*4*.*0*	*2*.*2*	*6*.*0*	*ND*	*2*.*3*	*ND*	
55741707	Ras-related protein Rab-8A	Exocytosis. Involved in polarized vesicular trafficking and neurotransmitter release.	3.2	ND	3.6	ND	ND	ND	
73987813	Synaptotagmin-like 2	Exocytosis. A key mediator of the docking of Rab27-bearing vesicles and organelles to the plasma membrane. Controls renal epithelial cell size through regulation of Rap—ezrin signaling independently of Rab27.	(2.6)	2	(0.6)	ND	ND	ND	[119]
61316260	Caveolin-2	Trancytosis. Acts as an accessory protein in conjunction with CAV1 in targeting to lipid rafts and driving caveolae formation. May act as a scaffolding protein within caveolar membranes.	(2.1)	ND	3.5	ND	ND	ND	
57101400	Ras-related protein Rab-3D	Exocytosis.	(2.0)	ND	3.9	ND	ND	ND	[120,121]

Accession	Name	Localization/Function-Clathrin-Dependent Endocytosis	OCLN N	OCLN C	CLD4 N	ZO-1 N	ZO-1 C	E-CAD	
345799451 345799449	Clathrin interactor 1	Clathrin dependent endocytosis. Stimulates clathrin assembly.	61.3	29.1	35.4	ND	ND	26.8	
73994175	SH3 domain-binding protein 4	Clathrin dependent endocytosis. Functions in transferrin receptor internalization.	2.5	2.9	(0.6)	ND	ND	(0.1)	
345780399	stromal membrane-associated protein 2	May play a role in clathrin-dependent retrograde transport from early endosomes to the trans-Golgi network.	(1.9)	2.7	ND	ND	ND	(1.0)	
73987780	Phosphatidylinositol-binding clathrin assembly protein	Clathrin-dependent endocytosis. Assembly protein recruiting clathrin and AP2 to coated-pit formation and clathrin-vesicle assembly.	ND	8.5	ND	ND	ND	11	
73994927 73966805	AP-1 complex subunit beta-1	Clathrin-dependent endocytosis. Mediates both the recruitment of clathrin to membranes and the recognition of sorting signals.	ND	ND	9.5	ND	ND	ND	

**Table 6:** Enriched cell adhesion proteins tagged by biotin ligase fused to occludin and claudin-4.

Accession	Name	Localization/Function-Cell Adhesion	OCLN N	OCLN C	CLD4 N	ZO-1 N	ZO-1 C	E-CAD	Reference
308081450	CD44 antigen precursor	Mediates cell-cell and cell-matrix interactions.	13.6	7.4	57.9	ND	7.4	(0.8)	
345803763	Leucine-rich repeat transmembrane protein FLRT2	May have a function in cell adhesion and/or receptor signaling.	7.4	2.8	8.6	ND	2.8	ND	
147898773	Carcinoembryonic antigen-related cell adhesion molecule 1 precursor	Membrane protein that mediate intercellular adhesion, proliferation, angiogenesis, apoptosis, immune responses, T cell cytotoxicity, differentiation, and polarization.	7.1	ND	34.5	ND	ND	3.5	[122]
74004342 345797211	Plakophilin-4	Plays a role as a regulator of Rho activity during cytokinesis. May play a role in junctional plaques.	6.8	3.8	4.2	ND	ND	3.0	
345793345	Integrin beta-1	Cell adhesion to exracellular matrix.	6.6	2.1	43.6	ND	2.1	ND	
147904030	Carcinoembryonic antigen-related cell adhesion molecule 28 precursor	Cell-cell adhesion molecule detected on leukocytes, epithelia, and endothelia.	6.6	2.7	15.3	ND	2.7	ND	
359318970 359318968	Protocadherin-1-like	Involved in cell-cell interaction and cell adhesion.	4.4	3.1	9.6	ND	3.1	ND	
345796205	Immunoglobulin superfamily member 11	Functions as a cell adhesion molecule through homophilic interaction.	3.8	2.9	(3.3)	4.8	2.9	ND	
359319033	Protein LAP2-like	Found in hemidesmosomes, which are cell-substrate adhesion complexes in stratified epithelia.	3.5	5.5	(1.0)	3	ND	ND	
50950141	Presenilin-1	Probable catalytic subunit of the gamma-secretase complex. May play a role in intracellular signaling and gene expression. Stimulates cell-cell adhesion though its association with the E-cadherin/catenin complex.	2.9	(1.6)	(1.6)	ND	ND	(0.8)	
345799149	Integrin alpha-2	Cell adhesion to exracellular matrix.	(2.6)	(0.6)	32.6	ND	ND	ND	
66472883	Podocalyxin precursor	Pro-adhesive protein, enhancing the adherence of cells to immobilized ligands, increasing the rate of migration and cell-cell contacts in an integrin-dependent manner.	(1.7)	(0.4)	17.48	ND	ND	ND	
73991135	Leucine-rich repeat transmembrane protein FLRT3	Cell adhesion and/or receptor signaling.	(1.2)	ND	6.2	ND	ND	ND	
50978890	Integrin beta-3 precursor	Cell adhesion to exracellular matrix.	ND	ND	10.2	ND	ND	ND	

**Table 7:** Enriched cytoskeletal proteins tagged by biotin ligase fused to occludin and claudin-4.

Accession	Name	Localization/Function-Cytoskeleton	OCLN N	OCLN C	CLD4 N	ZO-1 N	ZO-1 C	E-CAD
345804799 345804801	Brain-specific angiogenesis inhibitor 1-associated protein 2	Adapter protein that links membrane-bound small G-proteins to cytoplasmic effector proteins. Necessary for CDC42-mediated reorganization of the actin cytoskeleton and for RAC1-mediated membrane ruffling.	18.8	7.4	38.3	ND	ND	ND
359320122	Fermitin family homolog 2	Scaffolding protein that enhances integrin activation mediated by TLN1 and/or TLN2.	10.6	ND	13.2	ND	ND	ND
345780352	Septin-7	Filament-forming cytoskeletal GTPase involved in actin cytoskeleton organization.	10.1	7.4	16.3	ND	0.6	ND
345776847	Cdc42 effector protein 1	Organization of the actin cytoskeleton.	7.7	2.8	9.2	ND	ND	ND
73963313	Pleckstrin-2	Cytoskeletal arrangement and lamellipodia formation.	6.4	ND	ND	ND	ND	ND
73950452	MARCKS-related protein	Controls cell movement by regulating actin cytoskeleton homeostasis and filopodium and lamellipodium formation.	5.5	2.8	27.4	ND	ND	3.0
73955372	Profilin-1	Binds to actin and affects the structure of the cytoskeleton.	3.6	5.6	(2.7)	3.0	4.7	(0.6)
345780124	Neurabin-1	Binds to actin filaments and shows cross-linking activity.	3.3	ND	4.9	ND	ND	ND
73965092	Cdc42 effector protein 4	Organization of the actin cytoskeleton.	2.9	2.5	5.7	ND	ND	ND
73998521	Gamma-adducin	Membrane-cytoskeleton-associated protein that promotes the assembly of the spectrin-actin network.	2.7	2.3	5.5	ND	ND	ND
345789997	Erythrocyte membrane protein band 4.1-like 1	Major structural element of the erythrocyte membrane skeleton.	(2.5)	1.5	4.8	ND	ND	ND
73969750	EH domain-binding protein 1	May play a role in actin reorganization. Links clathrin-mediated endocytosis to the actin cytoskeleton.	(1.4)	4.0	ND	ND	ND	ND
345799052	PDZ and LIM domain protein 7	Adapter protein that, via its PDZ domain, localizes LIM-binding proteins to actin filaments.	ND	24.4	ND	ND	12.0	ND
74010709	Drebrin-like	Adapter protein that binds F-actin and DNM1, and thereby plays a role in endocytosis. Reorganization of the actin cytoskeleton and formation of cell projections via its interaction with WASL and COBL.	ND	7.6	ND	ND	12.9	ND
73952670	Ankyrin-3	Membrane-cytoskeleton linker.	ND	7.2	ND	ND	(0.6)	3.8
345794001	Phosphatase and actin regulator 4	Regulator of protein phosphatase 1 (PP1), actin-binding protein.	ND	1.7	ND	ND	ND	2.6
74003992	Tubulin beta-2A chain	Tubulin is the major constituent of microtubules.	ND	ND	9.4	ND	23.0	ND
345801304	BAI1-associated protein 2-like 1	Adapter protein. Involved in the formation of clusters of actin bundles. Plays a role in the reorganization of the actin cytoskeleton in response to bacterial infection.	ND	ND	9.2	ND	(0.8)	ND

**Table 8:** Enriched membrane proteins tagged by biotin ligase fused to occludin and claudin-4.

Accession	Name	Localization/Function-Membrane	OCLN N	OCLN C	CLD4 N	ZO-1 N	ZO-1 C	E-CAD	Reference
345794237	Plasmolipin	Appears to be involved in myelination. Receptor for McERV. Could also participate in ion transport.	37.9	12.2	35.3	ND	ND	ND	
345807298	SLIT and NTRK-like family, member 4	Slitrks have been implicated in mediating basic neuronal processes, full function unknown. Membrane protein.	29.6	23.3	21.1	4.0	ND	9.1	
73998757	Pleckstrin homology domain-containing family A member 1	Binds specifically to phosphatidylinositol 3,4-diphosphate. May recruit other proteins to the plasma membrane.	26.0	ND	ND	ND	ND	2.8	
114326335	Palmitoyltransferase ZDHHC5	Palmitoyl acyltransferase for the G-protein coupled receptor SSTR5.	10.7	9.6	4.5	ND	ND	ND	
345792339	Pleckstrin homology domain-containing family A member 5	Unknown function.	8.2	6.0	4.7	ND	ND	13.0	
345790319	Probable palmitoyltransferase ZDHHC20	Membrane enzyme.	7.8	1.7	8.1	ND	ND	ND	
73963551	Protein numb homolog	Membrane protein with a role in determination of cell fates during development and neurogenesis. May have a role in Notch signaling pathway.	5.7	ND	ND	ND	ND	6.5	
50979052	Annexin A13	Associated with the plasma membrane of crypt epithelial cells and villus enterocytes.	3.7	(1.0)	25.5	ND	ND	ND	
359322167	Paralemmin-1	Involved in plasma membrane dynamics, synapse maturation, spine formation and recruitment of AMPA-type glutamate receptors.	3.2	2.5	(2.5)	ND	ND	ND	[123]
73950747	Transmembrane protein 51	Membrane protein with unknown function.	3.2	2.2	5.3	ND	ND	ND	
73967304 345804993	Carboxypeptidase D	Enzyme. Releases C-terminal Arg and Lys from polypeptides.	3.1	(1.1)	4.5	ND	ND	0.6	
345793135	Junctophilin-1	Links the plasma membrane with the ER or sarcoplasmic reticulum.	(2.7)	3.4	(1.1)	ND	ND	0.8	
73987612	Basigin	Plays an important role in targeting the monocarboxylate transporters to the plasma membrane.	(2.6)	(0.7)	28.2	ND	ND	(0.4)	
345796185	Pleckstrin homology-like domain, family B, member 2	May play a role in acetyl-choline receptor (AChR) aggregation in the postsynaptic membrane.	(2.2)	2.1	(0.6)	ND	ND	ND	
345779148	Protein EFR3 homolog A	A component of a protein complex required for the synthesis of the phosphoinositide PtdIns4P, which has a variety of functions at the neural synapse. Localizes at the plasma membrane.	(1.2)	2.2	(1.5)	ND	ND	1.6	
73981384 345782539	Na(+)/H(+) exchange regulatory cofactor NHE-RF3	A scaffold protein that connects plasma membrane proteins and regulatory components, regulating their surface expression in epithelial cells apical domains.	ND	ND	8.7	ND	ND	ND	
345784192	Membrane-associated progesterone receptor component 2	Membrane steroid receptor.	ND	ND	5.3	ND	ND	3.9	
345781880	Syndecan-1	Cell surface proteoglycan that bears both heparan sulfate and chondroitin sulfate and that links the cytoskeleton to the interstitial matrix.	ND	ND	4.5	ND	ND	ND	

## Results and Discussion

### The Biotin Ligase Occludin and Claudin-4 Fusion Proteins Localize to Tight Junctions and Lateral Plasma Membranes

**Fig 1:**
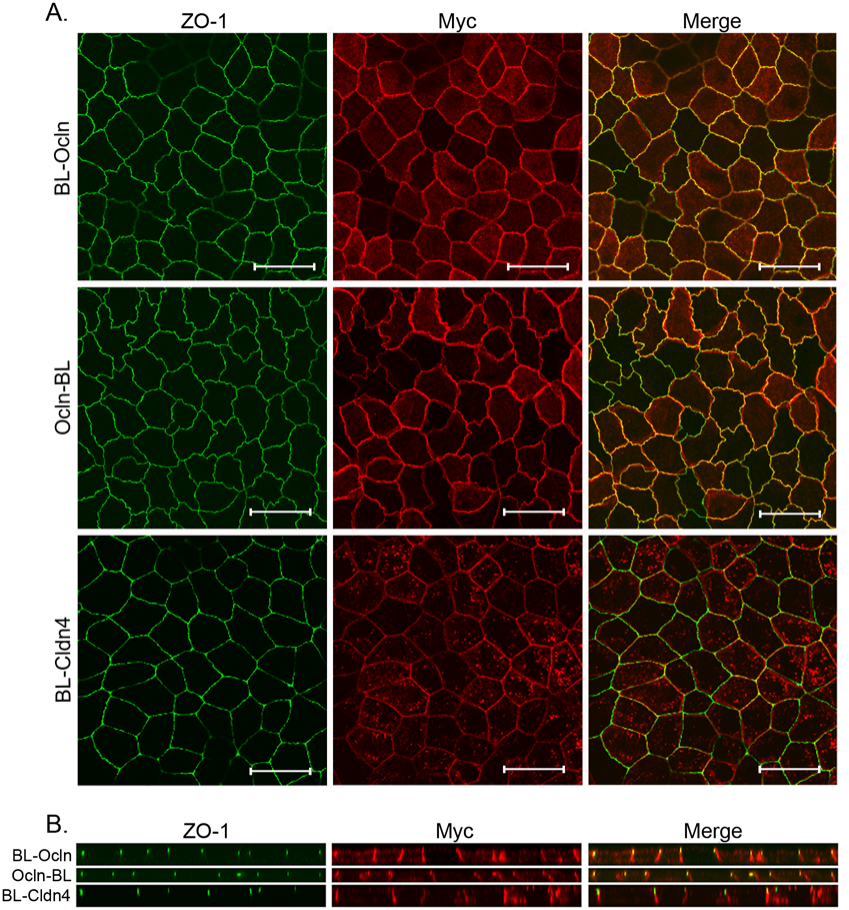
Ocln and Cldn4 biotin ligase fusion-proteins localize to the TJ and the lateral cell membrane. **A**. Both biotin ligase fused to the N terminus (BL-Ocln, myc) and C terminus (Ocln-BL, myc) of Ocln co-localized (*Merge*, *top right*, *middle right panel*) with ZO-1 (*top left and middle left panel*), although there is also some non-junctional immunofluorescence associated with the transgene. Biotin ligase fused to the N terminus of Cldn4 (BL-Cldn4, myc) partly co-localizes with ZO-1 (Merge, bottom right panel), and partly in the cytoplasm (Bottom middle panel). **B**. BL-Ocln, Ocln-BL and BL-Cldn4 (Myc signal middle panel) are distributed along the basolateral membrane. The two Ocln constructs also concentrate at the apical junction with ZO-1 (right top and middle panel). Bar, 20 microns.

In order to determine the spatial specificity of the labeling method we determined both the cellular localization of the fusion proteins and the subcellular patterns of biotinylated proteins. Unlike ZO-1 which is focused at the TJ, both claudins and Ocln also show variable localization to the lateral membrane [6,7,12,19,20,33,38,40]. As integral membrane proteins they are also expected to be near proteins in biosynthetic vesicular trafficking pathways [2,25,26,41–47]. As expected, en face immunofluorescent images of the TJ protein ZO-1 (Fig. 1A, left panels) and BL-Ocln, Ocln-BL and BL-Cldn4 fusion proteins (middle panels) reveals colocalization at TJs (right panels), as reported by myc epitope staining. The biotin ligase fusion proteins are also found to a variable extent in intracellular compartments. In contrast, we have previously shown that myc-tagged biotin ligase alone is diffusely distributed throughout the cells including the nucleus [10].

Similar to endogenous Ocln [6,12,20], Ocln biotin ligase fusion proteins are concentrated at the TJ, but there is also considerable lateral distribution of the transgenes (Fig. 1B, center panels). This may in part result from their over-expression. However, Ocln normally traffics to the TJ via the lateral cell membrane [16] and endogenous Ocln can be detected at this site with antibodies that recognize unphosphorylated Ocln [15]. These findings suggest that the use of biotin ligase Ocln transgenes to report proximal proteins should provide physiologically relevant information, both at the TJ and at the lateral membrane. Endogenous Cldn4 is localized both to the TJ and the lateral cell membrane with comparable immunofluorescent signal in cultured epithelial cells [34] and tissues [40]; thus the distribution of this transgene approximates that of the endogenous protein (Fig. 1B, bottom center panel). Even though the transgenes in Fig. 1 looks similar by myc staining, the proteins identified with mass spectrometry (MS) differ between the Ocln and Cldn4 biotin ligase fusion proteins (S2 and S3 Tables), and also differ from those identified using the laterally distributed E-cad biotin ligase fusion protein [11]. This suggests that biotin ligase proximity tagging reveals greater spatial resolution than is detectable by immunofluorescent localization.

**Fig 2:**
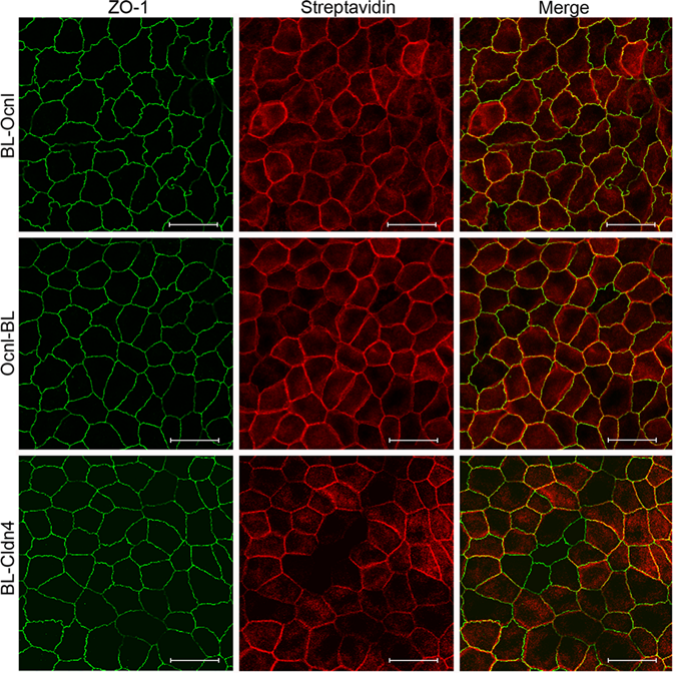
Streptavidin staining of biotinylated proteins reveals proteins at the TJ and lateral membranes. Biotin was added over night to MDCKII cells expressing the various biotin ligase fusion proteins. After wash, fixation, and block fluorescent streptavidin and ZO-1 primary antibody was added. Both biotin ligase fused to the N-terminus (BL-Ocln, myc) and C-terminus (Ocln-BL, myc) of Ocln tagged proteins near ZO-1 (Merge, top right and middle right panel). Biotin ligase fused to the N-terminus of Cldn4 (BL-Cldn4) also partly tags proteins co-localized with ZO-1 but possibly to a lesser extent than Ocln (*Merge*, *bottom right panel*). Although the majority of streptavidin stained proteins are distributed along the lateral plasma membrane at cell-cell contacts, there are also cytoplasmic staining (middle panel). Streptavidin staining of cells expressing biotin ligase alone reveal a much more diffusely distributed protein staining throughout all compartments of the cell [10]. Bar, 20 microns.

In order to verify that the biotinylated proteins were concentrated near the expressed fusion proteins, we incubated cell cultures expressing the biotin ligase fusion proteins with 50μM biotin for 16h, fixed the cells and stained them with fluorescent streptavidin. The results show similar, although slightly more diffuse distribution of streptavidin stained proteins (Fig. 2), as compared to the myc-fluorescent signal from BL-Ocln, Ocln-BL and Cldn4-BL (Fig. 1). We have previously demonstrated that fluorescent streptavidin stained cells expressing biotin ligase alone, after incubation with biotin, shows a diffuse staining pattern of biotinylated proteins all over the cell [10]. Given the overlapping distribution with ZO-1 as well as expression on the lateral cell membrane, we would expect proteins biotinylated by the fusion proteins to include TJ proteins also identified by ZO-1 as well as novel relevant lateral plasma membrane proteins and trafficking proteins.

### Coomassie-stained Protein Gels of Samples from MDCK II-cells Expressing Occludin and Claudin-4 Biotin Ligase Fusion Proteins Reveal Differences and Similarities in their Biotinylation Patterns

**Fig 3:**
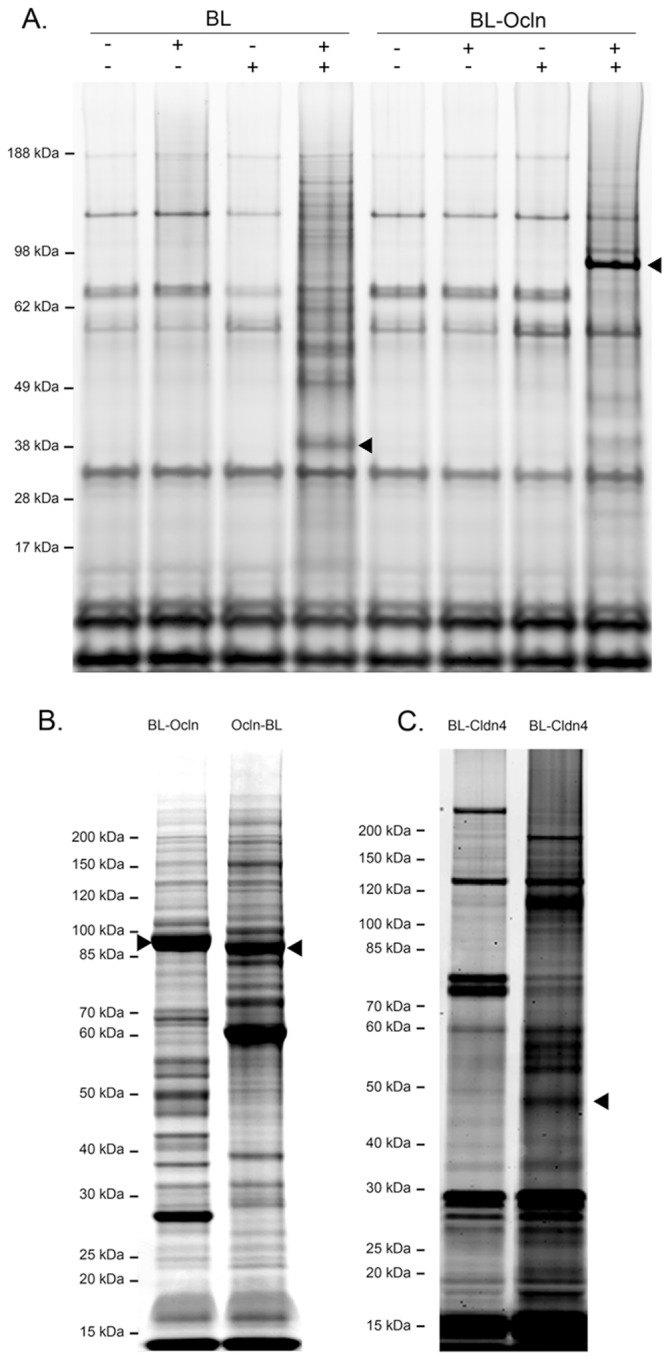
Coomassie-stained SDS-PAGE gels reveal that streptavidin-purified biotinylated proteins from different transgenes show differing protein patterns. **A**. Shown are proteins purified from cells expressing biotin ligase alone (*BL*), biotin ligase fused to the N terminus of Ocln (*BL-Ocln*). **B**. Proteins purified from the N- and C- terminus of Ocln (BL-Ocln and *Ocln-BL) with added* biotin. **C**. Shown are proteins purified from cells expressing biotin ligase fused to the N terminus of Cldn4 (*BL-Cldn4*), *with or without added* biotin. The positions of the transgenes are marked with *arrowheads*. Triplicate samples gave very similar protein patterns.

To determine whether there is specificity to the proteins labeled by the biotin ligase fusion proteins and purified on streptavidin resin, we first compared the pattern of purified proteins on SDS-PAGE gels from cells expressing biotin ligase alone (Fig. 3A, left panels) with proteins from cells expressing BL-Ocln (Fig. 3A, right panels). This analysis demonstrated that the pattern of recovered proteins was dependent on induction of the specific biotin ligase fusion protein and on the addition of biotin to the cell cultures. Protein bands from un-induced cells, and bands from cells in the absence of added biotin, likely reflect the presence of endogenously biotinylated proteins, including carboxylases [48]. The pattern of these bands of endogenously biotinylated proteins in all controls (un-induced cells without added biotin, induced cells without added biotin, and un-induced cells with added biotin) appear very similar (Fig. 3A). In contrast, the pattern of Coomassie-stained proteins from cells induced to express biotin ligase fusion proteins and incubated with exogenous biotin reveals novel protein patterns that varies among the different fusion proteins (for example, compare Fig. 3A, lanes 4 and 8). The marked reduction of many of the endogenously biotinylated protein bands in samples from cells with induced biotin ligase fusion-proteins, and added biotin, maybe due to competition for binding to the streptavidin resin.

In preparation for MS each sample was run on a SDS PAGE gel in duplicate lanes, one lane for visualization with Coomassie (Figs. 3B and 3C) and a second lane with the bulk of the sample for MS preparation (not shown). Replicate isolations from each fusion protein looked similar (not shown). All samples with induced biotin ligase fusion proteins and added biotin contained a large number of streptavidin extracted proteins, including self-biotinylated fusion proteins and biotin ligase alone (Fig. 3A, left panel), Ocln (Fig. 3A, right panel and Fig. 3B, right lane), and Cldn4 (Fig. 3C, right lane). The self biotinylation was stronger in both the Ocln biotin ligase fusion proteins as compared to biotin ligase alone and BL-Cldn4; this is likely due to the larger size of the Ocln fusion protein. The differing patterns visualized by Coomassie staining are consistent with each of these proteins tagging non-identical sets of proximal neighbors (S2 and S3 Tables).

### Proteomic Analysis Reveals Both Differences and Similarities in Protein Functional Categories Among Occludin and Claudin-4 Biotin Ligase Fusion Proteins

**Fig 4:**
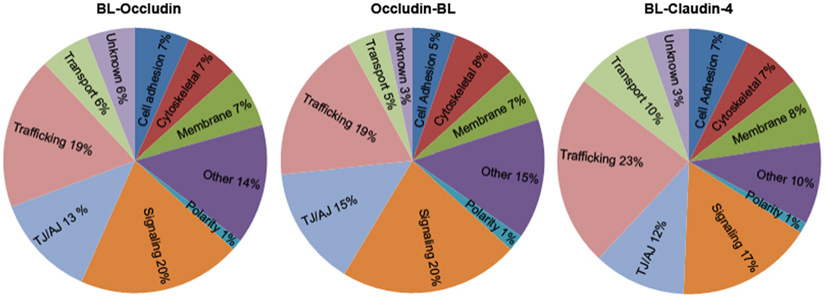
Functional analysis of top 150 enriched proteins recovered from cells expressing biotin ligase fusion proteins. Streptavidin-purified proteins identified by mass spectrometry from cells expressing biotin ligase fused to the N terminus of Ocln (*BL-Occludin*, *left*), the C terminus of Ocln (*Occludin-BL*, *middle)* or the N terminus of Cldn4 (*BL-Claudin 4*, *right*). Functional classification revealed similar distribution for the two Ocln constructs, although individual proteins within the functional groups trafficking-, signaling-, cell adhesion etc. vary, or are more or less abundant (higher or lower average normalized-PSM/OPN). Functional groups of proteins recovered from cells expressing biotin ligase fused to the N terminus of Cldn4 shows similar functional distribution of enriched proteins as both of the Ocln fusion proteins, although there are slightly more trafficking proteins tagged by BL-Cldn4. Enriched = ≥3-fold increase compared to biotin ligase alone (as determined by the average normalized PSM/OPN).

Triplicate MS analyses of proteins labeled with biotin by the biotin ligase fusion proteins, and purified by streptavidin binding, resulted in the identification of a large number of both expected and unexpected proteins. Abundantly tagged proteins by Ocln and Cldn4 biotin ligase fusion proteins included numerous TJ proteins including the coxsackievirus and adenovirus receptor homolog, ZO-1, partitioning defective 3 homolog and many claudins (Table 1). Other categories enriched around the Ocln- and Cldn4 biotin ligase fusion proteins were signaling, trafficking, membrane, cytoskeletal, cell-adhesion and transport proteins (Fig. 4). For comparison, Tables 1–8 present the most highly tagged proteins near the N- and C- termini of Ocln and the N-terminus of Cldn-4 along with previously published result for proteins near ZO-1 [10] and E-cad [11]. The full lists of Ocln and Cldn4 tagged proteins are presented in S2 Table.

To approximate the relative abundance of proteins recovered in the MS analyses, and to correct for overall recovery between experiments, the PSM value for each protein was normalized by dividing it with the total PSMs for that experiment. This value was then averaged between experiments and corrected for protein size by dividing with the theoretical observable peptide numbers (OPN) in the size range detectable by MS analysis [49]. As expected, the PSM values for the same protein varied among the three experiments. The mean coefficient of variation for the five most highly enriched proteins was 0.4 with a range between 0.1–1 (S5 Table). Inspection of the proteomic results revealed that some proteins were recovered at higher average normalized PSM/OPN than others. Some proteins were heavily tagged both in cells expressing biotin ligase alone and in Ocln and Cldn4 biotin ligase fusion-protein expressing cells; *examples include* transgelin-2 and PDZ and LIM domain protein 5. To focus on the proteins that were specifically tagged by Ocln and Cldn4 biotin ligase fusion proteins, we first removed all proteins that were less than 3-fold enriched compared to cells expressing biotin ligase alone. The full lists of enriched proteins around Ocln and Cldn4 are presented in S2 Table.

**Fig 5:**
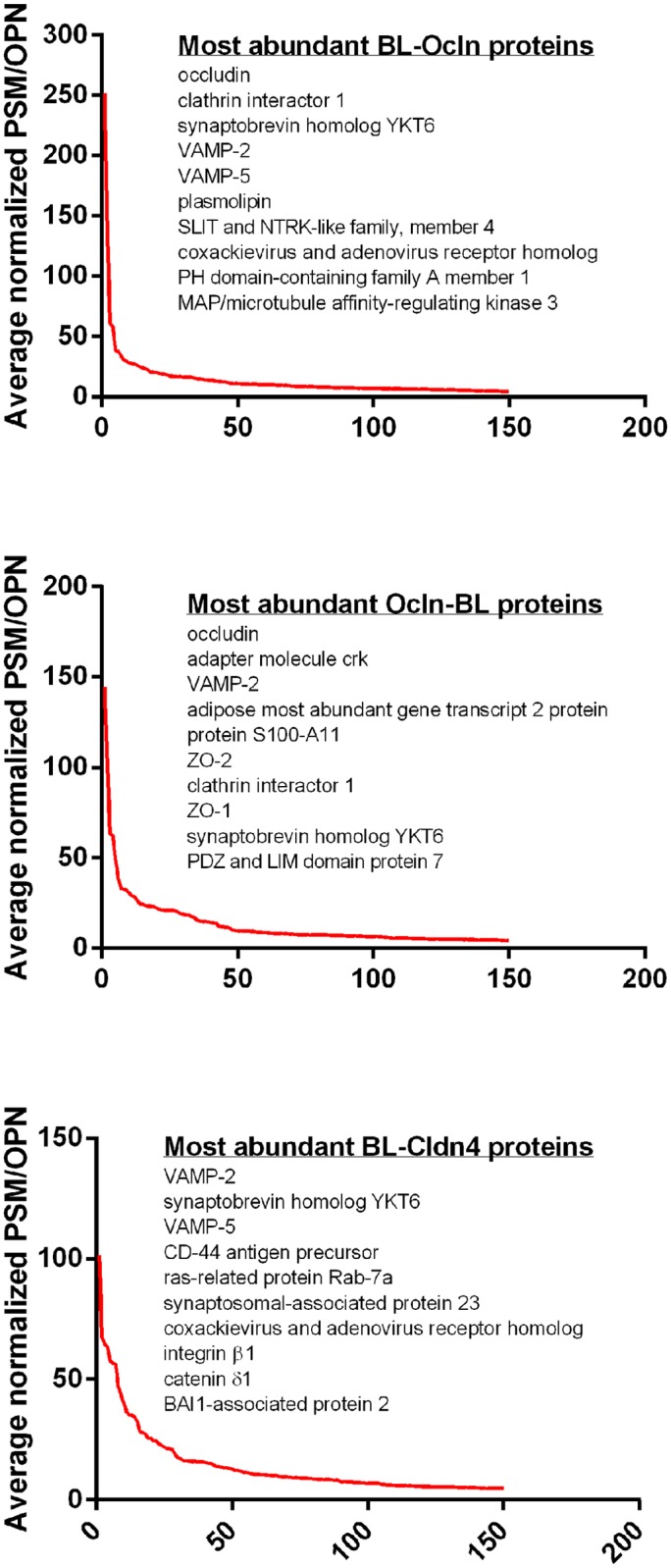
Relative abundance of proteins tagged by biotin ligase fusion proteins identified by mass spectrometry. The y-axis is proportional to the amount of protein recovered and was calculated as follows: PSMs from each of the three isolations were normalized (PSM for each protein/total PSMs for that isolation), these normalized PSMs were averaged between the three runs and then divided by the number of theoretical observable peptide number falling in the size range detectable by MS and this value multiplied by 1000. Proteins were ordered by this value (largest to smallest); points on the x-axis indicate individual unique proteins identified using the *Canis lupus familiaris* Ref Seq database. Plotted are the top 150 enriched (three-fold or above biotin ligase alone levels) proteins for each fusion-protein. The top 10 most enriched proteins for each fusion protein are listed.

Graphing the top 150 individual proteins in this enriched set from most abundant to least (by averaged normalized PSM/OPN) revealed that although many proteins were identified by MS, there were large quantitative differences in their recovery (Fig. 5). These differences could not only be a result of variability in spatial proximity to the biotin ligase fusion proteins, but also due to the number of available lysines and abundance and stability of the target proteins. Because proteins recovered with the highest average normalized PSM/OPN were likely to be the most biologically relevant, we chose to focus functional analysis on the top 150 most enriched proteins in each group (Figs. 4 and 5, S4 Table). Excluding self-biotinylated occludin, the top 10 most tagged proteins proximal to Ocln and Cldn4 include TJ proteins, trafficking proteins, such as VAMP2, VAMP5 and synaptobrevin homolog YKT6 and membrane proteins such as plasmolipin (Fig. 5). Of potential significance many of the top 10 proteins tagged by the Ocln and Cldn4 biotin ligase constructs have not previously been identified as TJ interacting proteins, and therefore deserve further study for potential roles in regulating TJ function.

When the top 150 enriched proteins proximal to Ocln and Cldn4 were analyzed by division into functional categories, as previously reported for BL-ZO-1 [10] and the E-cad-BL [11], the most abundant categories were those including signaling and trafficking proteins (Fig. 4). Ocln, Cldn4 and E-cad are all integral membrane proteins and the amino terminus of ZO-1 is positioned closer to the membrane than is the carboxyl terminus. Thus the proteome surrounding the N-terminus of ZO-1 could be expected to be more similar to those of Ocln and Cldn4. This is in contrast to the enrichment of cytoskeletal and “other” proteins enriched proximal to the C-terminus of ZO-1 [10], which is known to bind actin and other actin binding proteins [50]. We therefore conclude that it seems likely that similarities and differences in functional categories surrounding the different biotin ligase fusion proteins could be related to membrane proximity.

### Proteins Identified with Biotin Ligase Occludin and Claudin-4 Fusion Proteins Localize to Tight Junctions and Lateral Plasma Membranes

**Fig 6:**
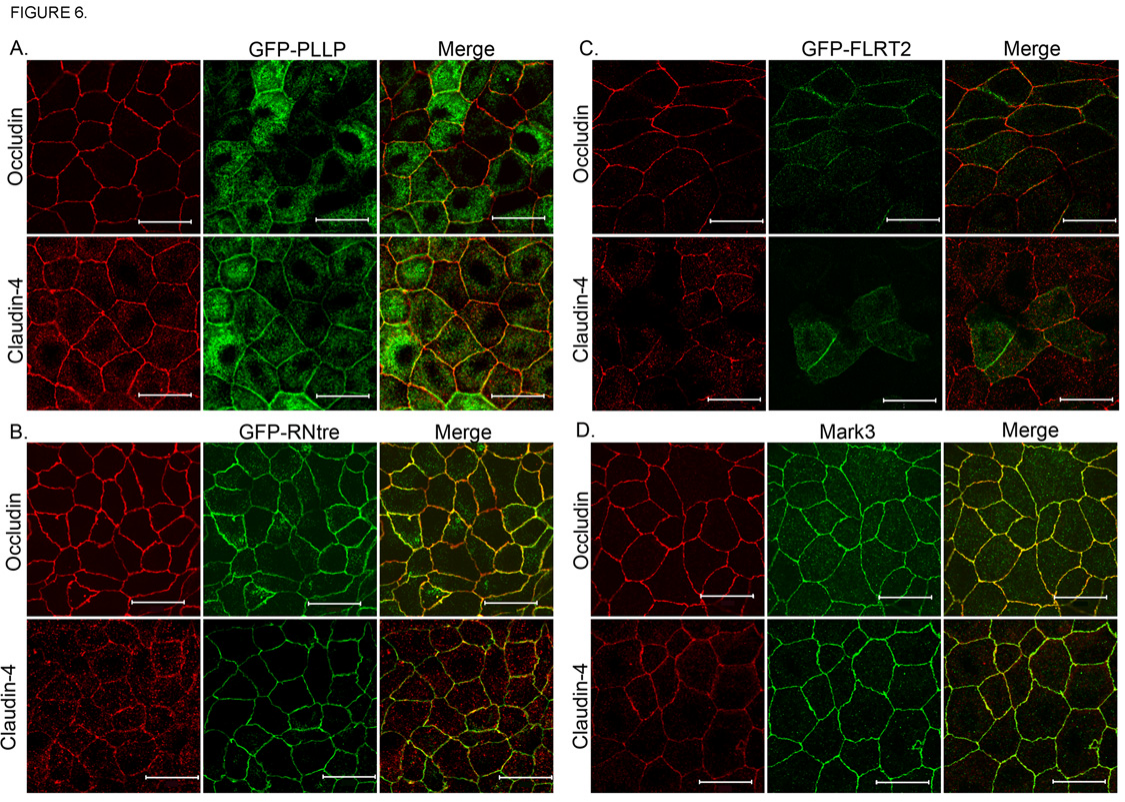
Cellular localization of proteins identified by proteomic analysis of proteins surrounding occludin and claudin-4. **A**. GFP-PLLP localizes diffusely in the cytoplasm and at cell-cell contacts (two middle panels). Co-localization with Ocln and Cldn4 appears to be at cell-cell contacts (two right panels). **B**. GFP-RNtre predominantly localizes to cell-cell contacts (two middle panels) where the co-localization with Ocln and Cldn4 occurs (two right panels). **C**. GFP-FLRT2 localizes diffusely in the cytoplasm and at cell-cell contacts (two middle panels).Co-localization with Ocln and Cldn4 appears to be at cell-cell contacts (two right panels). **D**. The majority of Mark3 localizes to cell-cell contacts but is also present in punctate structures in the cytoplasm (two middle panels). Ocln and Cldn4 co-localize with Mark3 at cell-cell contacts (two right panels). Bar, 20 microns (x63 oil objective).

To verify that proteins biotinylated by the biotin ligase fusion proteins and identified with mass spectrometry co-localize with endogenous occludin and claudin-4, we used immunofluorescent techniques to visualize several novel proteins in cultured MDCK II cells, namely plasmolipin, RNtre, FLRT2 and Mark3. The multi-pass transmembrane protein plasmolipin (PLLP) was highly enriched around both Ocln and Cldn4. GFP-tagged PLLP co-localizes with Ocln and Cldn4 at the TJ and along the basolateral plasma membrane in MDCK II cells (Fig. 6A, S1 Fig.). GFP-PLLP is also diffusely distributed in the cytoplasm (Fig. 6A, S1 Fig.). The trafficking protein USP6 N-terminal-like protein, also called: related to the N-terminus of tre (RNtre) was identified with mass spectrometry in the neighborhoods surrounding Ocln, Cldn4, ZO-1 and E-cad. GFP-RNtre co-localizes with both Ocln and Cldn4 at the lateral plasma membrane and TJ (Fig. 6B, S2 Fig.). GFP-tagged Leucine-rich repeat transmembrane protein FLRT2 (FLRT2), a protein involved in cell adhesion and or receptor signaling, was localized diffusely in the cytoplasm as well as co-localized with Ocln and Cldn4 along the basolateral plasma membrane and the apical TJ (Fig. 6C, S3 Fig.). Rabbit antibody to MAP/microtubule affinity-regulating kinase 3 (Mark3), the most highly enriched kinase proximal to the N-terminus of Ocln and also enriched around Cldn4, ZO-1 and E-cad, revealed diffuse cytoplasmic as well as distinct apical TJ staining in MDCKII cells. Mark3 was co-localized with Ocln at the apical region of the lateral cell membrane and with both Ocln and Cldn4 at the basolateral membrane below the TJ (Fig. 6D, S4 Fig.).

### Kinases, Segment Polarity Homologs, Eph/Ephrin and Other Signaling-Associated Proteins are Enriched in the Neighborhoods Around Occludin and Claudin-4

Signaling proteins, including kinases, phosphatases, signaling adapters/scaffolds and membrane receptors were the most highly enriched group around both the N- and the C-terminus of Ocln and second largest group enriched around the N-terminus of Cldn4 (Fig. 4, Table 2 and Table 3). Many of the signaling proteins found in this study have been shown to play important roles in regulating cytoskeleton reorganization, cell polarity, cell adhesion and cell fate (e.g. differentiation, proliferation and apoptosis), processes which all previously has been shown to be of important relevance to the TJ [51–54]. Some of these proteins were also identified in proteomic screens using biotin ligase fused to ZO-1 and/or E-cad, but many others were found enriched uniquely in the Ocln and/or the Cldn4 proteomes. For example, among the most abundant proteins identified proximal to Ocln was adapter molecule CRK, enriched both around the N- and the C-terminus (av n-PSM/OPN of 24.6 and 71.7 respectively). CRK was also previously shown to be enriched in the neighborhoods of the N- and C-terminus of ZO-1 (av n-PSM/OPN of 17.6 and 51.6 respectively) and the C-terminus of E-cad (av n-PSM/OPN of 22.1), however it is not enriched around Cldn4 (Table 2). CRK is reported to interact with mitogen-activated protein kinase kinase kinase kinase 5 [55], which was also highly enriched in the BL-Ocln proteome (av n-PSM/OPN of 23.3) but not the Ocln-BL and BL-Cldn4 (Table 3). Another example of difference in the biotin ligase fusion protein proteomes is the finding that all three members of the adaptor protein family DVL-1, -2 and-3 are identified as proximal proteins to ZO-1, E-cad and Ocln at comparable abundances, but was not enriched in the Cldn4 proteome (Table 2). DVL-1 has previously been associated with cell-cell junctions [56].

In contrast, some signaling proteins were identified as proximal to both Ocln and Cldn4 but were not found in the ZO-1 proteome. For example, Eph/Ephrin signaling proteins, involved in bidirectional signaling responsible for modulation of cell adhesion and developmental processes [57], were enriched around Ocln and Cldn4 but not ZO-1 (Table 2 and Table 3). The interaction between Cldn4 and Eph-A2 has previously been shown to lead to tyrosine phosphorylation of Cldn4, in turn resulting in increased paracellular permeability [58]. In addition, Cldn4 has also been shown to interact with ephrin-B1, leading to tyrosine phosphorylation of ephrin-B1 which affected intercellular adhesion [59]. Ephrin-B1 was enriched around both ends of Ocln and was found at the highest abundance at the N-terminus of Cldn4 (Table 2), whereas ephrin type-A receptor 1 (EPHA1) and EPHA2 were only enriched around Cldn4 (Table 3). Ephrin-B1 and EPHA1 were previously shown to be enriched around E-cad [11], although at lower abundances than cldn4, whereas no members of this family were detected in the enriched lists of ZO-1 [10] (Table 2 and Table 3). Similarly, members of the src family of protein tyrosine kinases including src, lyn and yes were enriched at the highest abundance in the Cldn4 proteome. They were also enriched in the Ocln, but not in the ZO-1 and E-cad, proteomes (Table 3). Yes and src have both been previously associated with Ocln [60–62].

Some signaling proteins were enriched only around Cldn4. One example is tumor-associated calcium signal transducer 2 (TROP-2; Table 2), a single-pass transmembrane glycoprotein belonging to the EPCAM family. Loss of TROP2 function is associated with corneal dystrophy and knockdown of TROP2 in epithelial cells has been shown to lead to deceased TER and possibly to prevent proper TJ localization of claudins 1 and 7 [63].

In general, Ocln is near the largest number of signaling proteins, however some of these signaling proteins were also found in the neighborhood around Cldn4, ZO-1, and E-cad suggesting functional overlaps (Table 2 and Table 3), [10,11].

### Endocytic Proteins are the Most Common Trafficking Proteins Near Both Occludin and Claudin-4

Trafficking proteins was the largest functional group of proteins enriched around the N-terminus of Cldn4 and second largest around both the N- and the C-termini of Ocln (Fig. 5). The trafficking proteins were further sub-divided into four separate groups, endocytosis, clathrin-dependent endocytosis, ER/Golgi and exocytosis/trancytosis (Table 4 and Table 5). The majority of trafficking proteins were found to be within the endocytosis subgroup, followed by ER/Golgi, exocytosis/transcytosis and clathrin-dependent endocytosis. The most highly enriched trafficking proteins around Ocln and Cldn4 were the endocytic SNARE proteins synaptobrevin homolog YKT6-like (YKT6), clathrin interactor 1 (CLINT1), vesicle-associated membrane protein 2 (VAMP2) and VAMP5 (Table 4 and Table 5). A VAMP2 interacting protein, syntaxin 6 [64], was also enriched around Ocln and Cldn4 although at lower abundances (Table 4). VAMP5 was previously shown to be enriched around E-cad and the N-terminus of ZO-1, potentially indicating a more general role for VAMP5 in trafficking of TJ associated proteins (Table 4). YKT6 has not only been shown to have a function in endocytosis [65], but has also been linked to ER-Golgi transport [66] which may mean that the high biotinylation of this protein could have occurred during protein synthesis of the biotin ligase fusion proteins.

Many members of the Rab GTPase family were enriched around Ocln- and Cldn4 biotin ligase fusion proteins. The most highly tagged Rab GTPases by Ocln and Cldn4 biotin ligase fusion proteins were Rab-5b, Rab-7a, Rab-8a, Rab-10 and Rab-23 (Table 4). Only Rab-7a, a Rab that controls vesicular membrane traffic to late endosomes and lysosomes as well as maturation of phagosomes and autophagic vacuoles, was also enriched around E-cad [11], but at a significantly lower abundance (Table 4). Rab7 has previously been shown to co-localize with Cldn4 and Ocln in internalized vesicular structures [42,67].

Several members of the Rab 11 family interacting proteins (RABFIP) were highly associated with both the N- and the C-termini of Ocln and only RAB11FIP1 was also associated with Cldn4 (Table 4). More specifically, RAB11FIP1, RAB11FIP2 and RAB11FIP5 were highly associated with both Ocln biotin ligase fusion proteins. RAB11FIP2 phosphorylation has previously been shown to regulate polarity and localization of TJ proteins in MDCKII cells [68]. Both the phosphomimetic and WT RAB11FIP2 overexpression resulted in recruitment of claudin-1 and claudin-2 to TJ whereas the phosphorylation mutant failed to recruit Cldn4 and Ocln. The enrichment of RAB11FIP around Ocln, supports the idea that Ocln delivery and recycling is important to maintain and regulate epithelial paracellular barrier function both during steady state and epithelial wound healing [24,47].

Taken together the trafficking proteins identified in our proteomic study of Ocln and Cldn4 neighboring proteins, combined with previously published ZO-1 and E-cad data [10,11], indicate that the transmembrane barrier sealing proteins are more highly associated with trafficking proteins than the intracellular TJ scaffold ZO-1. This finding could possibly mean that the regulation of these transmembrane proteins is more dependent upon efficient turnover than ZO-1, e.g. that they are being delivered, removed and recycled to the plasma membrane (or degraded in lysosomes) at higher rates. Of note for future studies, none of the most highly enriched trafficking proteins found in this study has so far been described in the TJ literature.

### Cell Adhesion Proteins are Enriched Around Occludin and Claudin-4

Complex cell-cell and cell-matrix interactions play crucial roles in mediating and regulating many processes, including cell migration, tissue homeostasis, wound healing, and tumorigenesis. CD44 antigen precursor, a protein that has been shown to play a role in both cell-cell and cell-matrix adhesion and to regulate TJ assembly and barrier function [69], was the most highly enriched within the cell adhesion functional category surrounding both Ocln and Cldn4, with the strongest association at the N-terminus of Cldn4 (Table 6). In the cell-matrix adhesion category integrin β1- and α2 were enriched around both Ocln and Cldn4, and β3 only around Cldn4. Overall, the integrins were more highly enriched in the Cldn4 neighborhood as compared to Ocln (for example compare av n-PSM/OPN of 43.6 at Cldn4 N-terminus to 6.6 and 2.1 at the N- and the C-terminus of Ocln). Although no studies thus far have shown direct interactions between Cldn4 and integrins, a number of other claudins have. For example, *β*1-integrin-mediated adhesion of brain endothelial cells to the surrounding ECM is critical for stabilizing claudin-5 at blood brain barrier (BBB) TJ, and to maintain BBB integrity [70]. Complexes of claudin-7, integrin α2, and claudin-1 have also been shown to be of importance for normal epithelial basolateral compartments of intestines [71].

Two members of the Leucine-rich repeat transmembrane protein (FLRT) FLRT2 and FLRT3, believed to be involved in cell adhesion and/or cell signaling [72–75], were both enriched around Cldn4 and FLRT2 was also enriched around the N- and the C-terminus of Ocln (Table 6). FLIRT2 knockout has been shown to lead disruptions to the epicardial cell layer preventing fully formed cell-cell junctions [76].

### Cytoskeletal, Membrane, Transport, Other, and Unknown- Proteins are also Enriched in the Neighborhoods around Occludin and Claudin-4

Apart from the predominant functional categories, e.g. signaling and trafficking proteins, several other groups of proteins were found in Ocln and Cldn4 proteomes including cytoskeletal, membrane, transport, other proteins and proteins of unknown function.

TJ proteins are connected to the actin-cytoskeleton via ZO-1 and other scaffolding proteins such as spectrin and erythrocyte membrane protein band 4.1 [77–79] (S2 Table). There are also other proteins interacting with the TJ that regulate cytoskeleton reorganization through intracellular signaling pathways and transcription regulation such as CDC42 and BAI1-associated protein 2 [80–83], (Table 7). Interestingly, even though the percentage of total enriched proteins identified as cytoskeletal around ZO-1 was higher, especially the C-terminus of ZO-1 [10], only 5 of the 19 cytoskeletal proteins found around Ocln and/or Cldn4 in this study were also identified around ZO-1 (Table 7), indicating different neighboring cytoskeletal partners. In addition, the percentage of cytoskeletal proteins enriched around E-cad was similar to that of both Ocln and Cldn4, however only three proteins were identical [11]. Future studies are needed to understand the protein interactions regulating the interplay between the TJ proteins and the actin cytoskeleton.

Many membrane proteins were biotinylated by Ocln and Cldn4 biotin ligase fusion proteins (Table 8). Among the most highly enriched membrane proteins around the N-terminus of Ocln was plasmolipin (PLLP) (av n-PSM/OPN of 37.9). PLLP was also enriched, but at lower abundance, at the C-terminus of Ocln and the N-terminus of Cldn4. PLLP is a MARVEL-domain containing tetraspan protein with sequence similarities with Ocln, tricellulin and marvel D3 [84]. PLLP has been localized both to apical and basolateral plasma membranes in epithelial cells in a variety of tissues [85]. The most highly enriched membrane protein around Cldn4 was basigin (CD147; av n-PSM/OPN of 28.2). Basigin is a transmembrane glycoprotein involved in embryonic development [86], extracellular matrix metalloproteinase (MMP) induction [87] and promotion of epithelial-mesenchymal transition (EMT) [88]. Basigin has been shown to have a basolateral membrane localization in thyroid epithelial cells (FRT) and various basigin mutants transfected into MDCK II cells also localize to the basolateral membrane [89], indicating a potential co-localization and a possible functional interaction with other lateral membrane proteins such as Cldn4. Basigin interaction with Cldn4 has not been described, but clustering of basigin with galectin-3 results in MMP9 release initiating cell-cell disassembly and redistribution of Ocln through its N-terminal domain in corneal epithelial cells [90].

The group of proteins designated to “other” function in Fig. 4 and S4 Table was in general low on the top 150 enriched proteins lists surrounding Ocln and Cldn4. A couple of exceptions included adipose most abundant gene transcript 2 protein (APM2) and breast carcinoma-amplified sequence 1 (BCAS1). BCAS1, a protein that is increased in for example breast cancer and is found in cytoplasmic vesicular structures [91], was enriched around both Ocln biotin ligase fusion proteins and Cldn4, with the highest abundance at the N-termini of Ocln and Cldn4 (S4 Table). AMP2, also implicated to have a role in cancer [92], was highly enriched around the C-terminus of Ocln (Fig. 5, S4 Table), but also at the N-terminus of Ocln.

Two members of the unknown protein group were enriched around many of the biotin ligase constructs tested in our lab; these are sickle tail protein homolog (SKT) and protein FAM83F. SKT was the most highly enriched around E-cad (av n-PSM/OPN of 31.7), but was also enriched around Ocln and ZO-1 (S4 Table). FAM83F was present with the strongest abundance at the N-terminus of Cldn4, but it was also enriched in the neighborhoods of Ocln, ZO-1 and E-cad (S4 Table).

Taken together, even though most proteins identified in the Ocln and Cldn4 proteomes were signaling, trafficking and known TJ/AJ interacting proteins, our data shows that some proteins assigned to other functional categories were also present at high abundance and deserve further investigation for a role in junction regulation.

### Conclusion

The proteins identified by the Ocln and Cldn4 biotin ligase fusion proteins in this study should provide a resource for further understanding the organization and function of tight junctions. When prioritizing proteins for further study it seems appropriate to start with those tagged at the highest level. Alternatively, proteins falling in functional categories highly enriched around Ocln and Cldn4 for example, signaling or endocytic proteins could provide new insights into these functions near tight junctions. Although the many signaling, trafficking and cytoskeletal proteins identified are unlikely to be unique to tight junctions, their identification in this screen suggests that they could play important roles associated with this complex structure. Finally, comparison between proteins tagged by biotin ligase fusion proteins of Ocln and Cldn4, and those identified in our previous studies of E-cad and ZO-1 [10,11], should allow identification of sets of tight- and adherens junction proteins and their compartmentalization.

## Supporting Information

S1 Fig Z-axis localization of plasmolipin, a protein identified by proteomic analysis of proteins surrounding occludin and claudin-4. GFP-PLLP localizes along the basolateral plasma membrane and diffusely in the cytoplasm (second and fifth panel). Co-localization with Ocln and Cldn4 can be seen along the lateral membrane (third and sixth panel. Cells were imaged with x63 oil objective.
(TIF)

S2 Fig Z-axis localization of RNtre, a protein identified by proteomic analysis of proteins surrounding occludin and claudin-4. GFP-RNtre predominantly localizes to the apical side of the basolateral plasma membrane (second and fifth panel) where the co-localization with Ocln occurs (third panel). Cldn4/RNtre co-localization also occurs at the basolateral membrane, but below the apical region. Cells were imaged with x63 oil objective.
(TIF)

S3 Fig Z-axis localization of FLRT2, a protein identified by proteomic analysis of proteins surrounding occludin and claudin-4. GFP-FLRT2 localizes diffusely in the cytoplasm and along the basolateral plasma membrane (second and fifth panel). Co-localization with Ocln and Cldn4 is present along the lateral membrane (third and sixth panel). Cells were imaged with x63 oil objective.
(TIF)

S4 Fig Z-axis localization of Mark3, a protein identified by proteomic analysis of proteins surrounding occludin and claudin-4. Mark3 predominantly localizes to the apical region of the lateral plasma membrane, but is also present in punctate structures in the cytoplasm (second and fifth panel). Ocln and Cldn4 co-localize with Mark3 at apical TJ (third and sixth panel). Cells were imaged with x63 oil objective.
(TIF)

S1 Table Infusion primers. Sense and antisense infusion primers for mycBioID, EGFP-RNtre, EGFP-FLRT2 and EGFP-PLLP are listed.
(XLSX)

S2 Table All proteins identified around BL-Ocln, Ocln-BL and BL-Cldn4. Numbers in the columns for biotin ligase constructs are average normalized PSM/OPNx1000. Ribosomal proteins can be found below each column respectively.
(XLSX)

S3 Table Enriched proteins identified around BL-Ocln, Ocln-BL and BL-Cldn4. Numbers in the columns for biotin ligase constructs are average normalized PSM/OPNx1000 from 3-fold enriched proteins compared to the biotin ligase alone [11]. Ribosomal proteins can be found below each column respectively.
(XLSX)

S4 Table Functional categories of enriched proteins identified around BL-Ocln, Ocln-BL and BL-Cldn4. Numbers in the columns for biotin ligase constructs are average normalized PSM/OPNx1000 from 3-fold enriched proteins (S3 Table) compared to the biotin ligase alone [11]. Numbers in parenthesis shows that the protein is enriched, however not in the top 150. Not detectable (ND) means that a protein is not enriched. Data from ZO-1 and E-cad are taken from previously published data [10,11]. If a reference is not listed in the far right column, UniProt is the source of protein localization/function. *Italic* proteins fall into more than one functional category, for example exocytosis and endocytosis.
(XLSX)

S5 Table Coefficient of variation for top five enriched proteins. Normalized PSM values were divided by observable peptide number (OPN) for each of the three individual experiments and fusion proteins. Coefficient of variation (C_v_) was calculated by dividing the standard deviation (SD) with the mean.
(XLSX)
