# Supplementary figures and images for: Proteomic Analysis of Proteins Surrounding Occludin and Claudin-4 Reveals Their Proximity to Signaling and Trafficking Networks
Source: PLoS One. 2015 Mar 19;10(3):e0117074. doi: 10.1371/journal.pone.0117074 (PMC4366163; doi:10.1371/journal.pone.0117074)

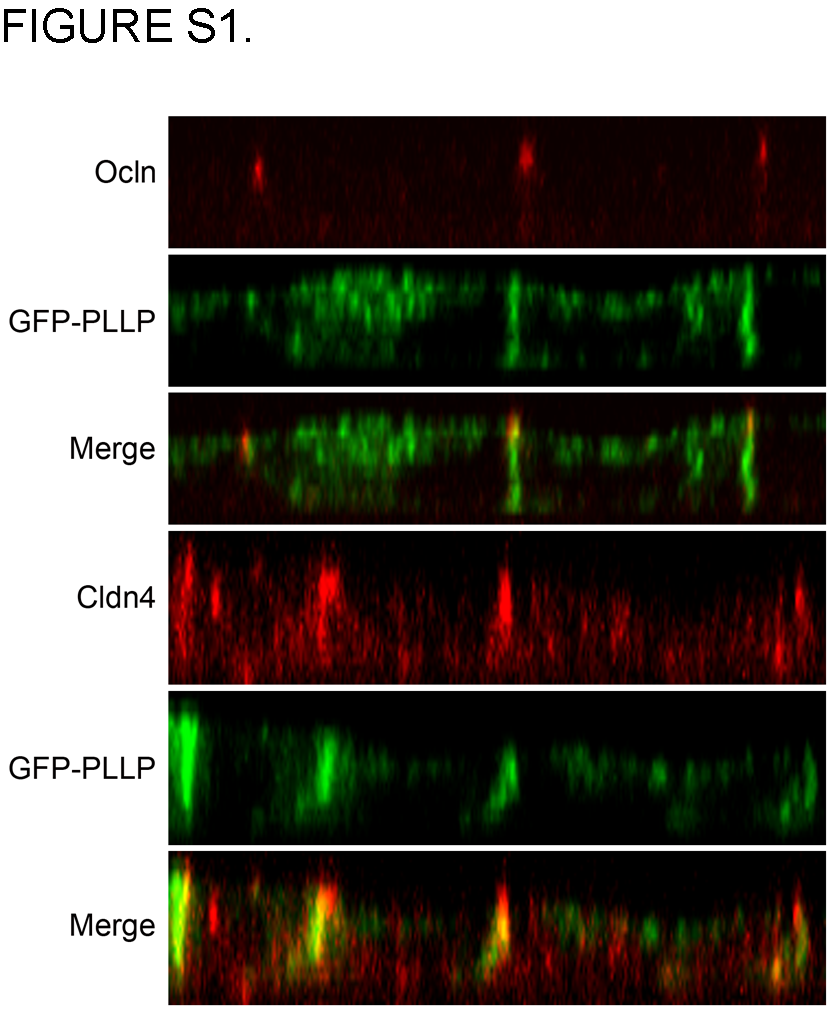

Supplement: S1 Fig — GFP-PLLP localizes along the basolateral plasma membrane and diffusely in the cytoplasm (second and fifth panel). Co-localization with Ocln and Cldn4 can be seen along the lateral membrane (third and sixth panel. Cells were imaged with x63 oil objective. (TIF) [file pone.0117074.s001.tif]

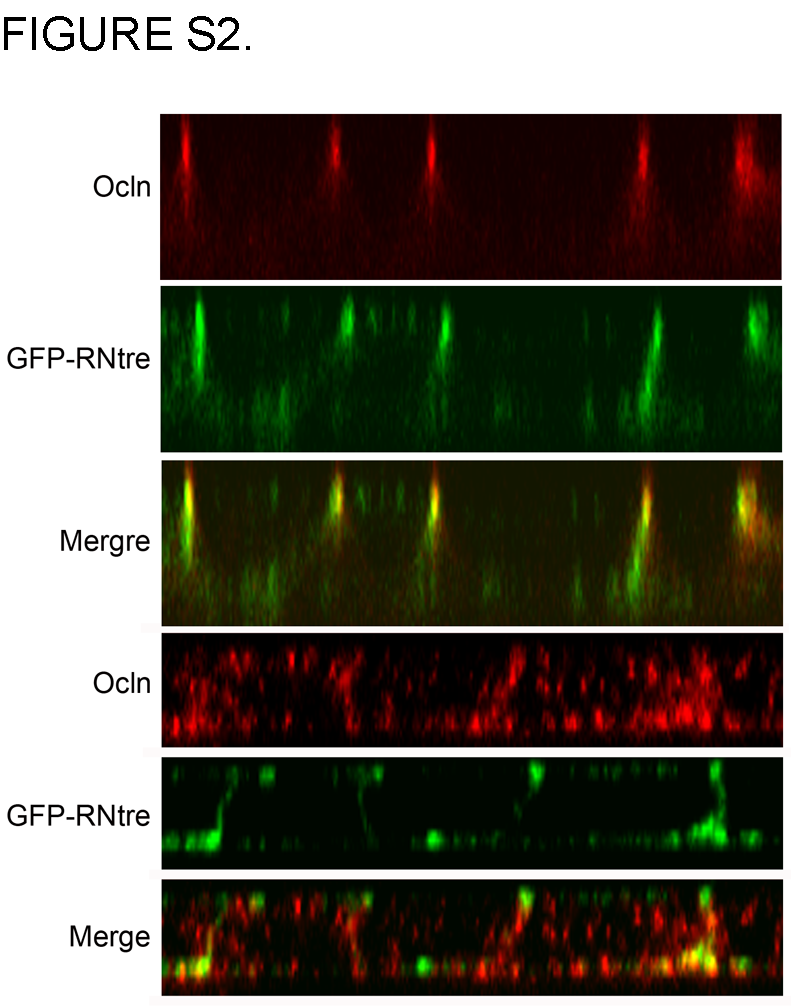

Supplement: S2 Fig — GFP-RNtre predominantly localizes to the apical side of the basolateral plasma membrane (second and fifth panel) where the co-localization with Ocln occurs (third panel). Cldn4/RNtre co-localization also occurs at the basolateral membrane, but below the apical region. Cells were imaged with x63 oil objective. (TIF) [file pone.0117074.s002.tif]

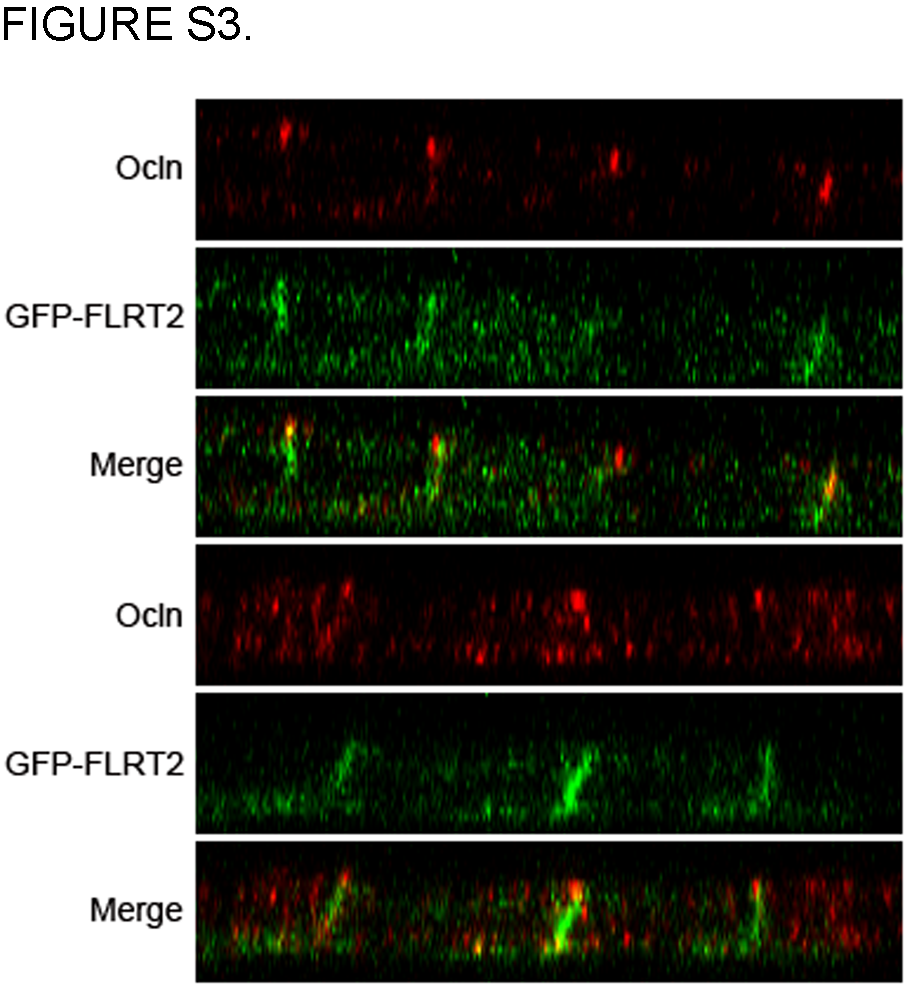

Supplement: S3 Fig — GFP-FLRT2 localizes diffusely in the cytoplasm and along the basolateral plasma membrane (second and fifth panel). Co-localization with Ocln and Cldn4 is present along the lateral membrane (third and sixth panel). Cells were imaged with x63 oil objective. (TIF) [file pone.0117074.s003.tif]

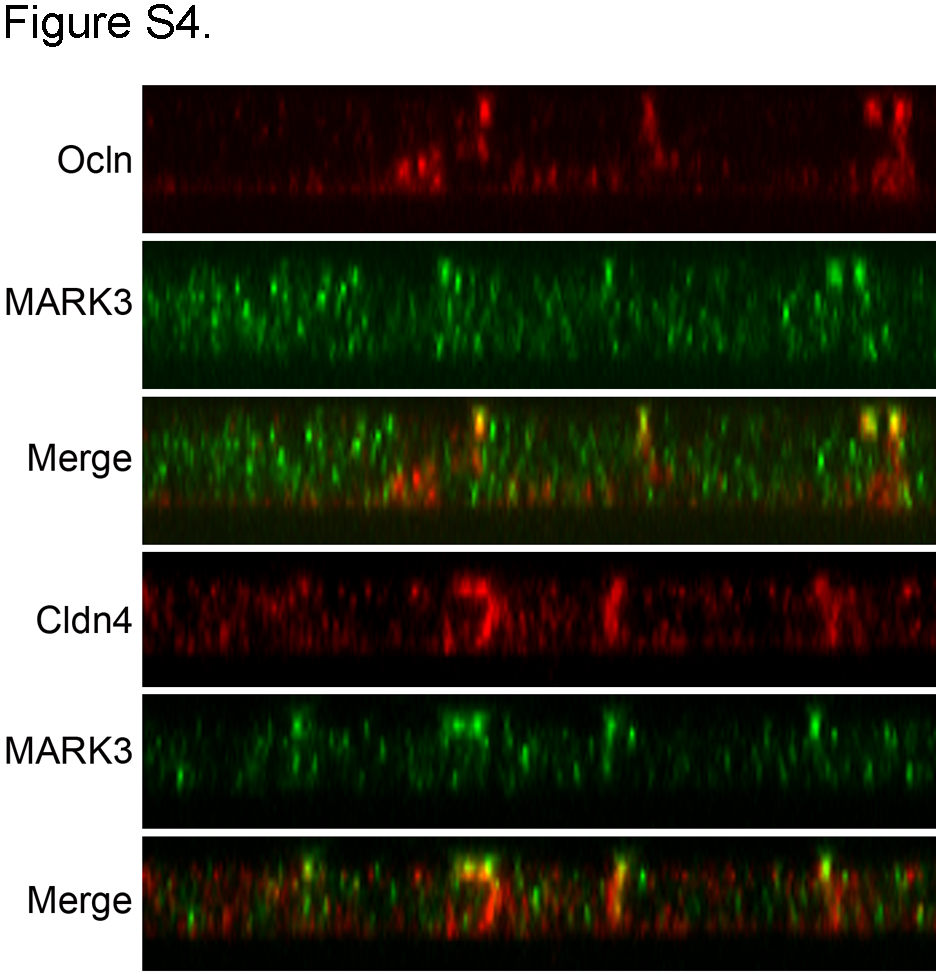

Supplement: S4 Fig — Mark3 predominantly localizes to the apical region of the lateral plasma membrane, but is also present in punctate structures in the cytoplasm (second and fifth panel). Ocln and Cldn4 co-localize with Mark3 at apical TJ (third and sixth panel). Cells were imaged with x63 oil objective. (TIF) [file pone.0117074.s004.tif]
